# Changes in the Fatty Acid Composition and Antioxidant Properties in Mono-Protein Commercial Dry Dog Foods During Storage

**DOI:** 10.3390/molecules30173524

**Published:** 2025-08-28

**Authors:** Jagoda Kępińska-Pacelik, Wioletta Biel, Robert Witkowicz, Piotr Micek, Ewa Piątkowska, Aleksandra Patla

**Affiliations:** 1 Department of Monogastric Animal Sciences, Division of Animal Nutrition and Food, West Pomeranian University of Technology in Szczecin, Klemensa Janickiego 29, 71-270 Szczecin, Poland; wioletta.biel@zut.edu.pl; 2 Department of Agroecology and Crop Production, University of Agriculture in Krakow, Mickiewicza 21, 31-120 Krakow, Poland; robert.witkowicz@urk.edu.pl; 3 Department of Animal Nutrition and Fisheries, University of Agriculture in Krakow, al. Mickiewicza 24/28, 30-059 Krakow, Poland; piotr.micek@urk.edu.pl (P.M.); aleksandra.knapik@urk.edu.pl (A.P.); 4 Department of Human Nutrition and Dietetics, Faculty of Food Technology, University of Agriculture in Krakow, Balicka 122, 30-149 Krakow, Poland

**Keywords:** dry dog food, nutritional composition, oxidative stability, shelf life

## Abstract

Dogs are the most popular companion animals in Europe, with an estimated population of 106 million in households. Commercial dry dog foods are formulated to meet specific nutritional requirements and ensure safety during storage, often through the addition of preservatives to extend shelf life. This pilot study investigated the antioxidant properties and changes in the fatty acid composition during storage in six mono-protein (containing only one source of animal protein) dry dog foods. These findings might contribute to a better understanding of the long-term nutritional stability of commercial dry dog foods and their potential implications for canine health. Changes in chemical composition, fatty acid profile, and antioxidant properties were analyzed immediately after opening the packages and after 3 and 6 months of storage. Significant alterations (*p* ≤ 0.05) were observed in nutrient content, particularly crude fat level (decrease from 18.37 g/100 g DM to 16.87 g/100 g DM after 6 months), as well as saturated and unsaturated fatty acids. Antioxidant properties, assessed via DPPH, FRAP, and ABTS, fluctuated over the storage period. Principal component analysis identified distinct patterns in nutrient and antioxidant profiles, highlighting the impact of storage duration and initial food composition on the stability of nutritional and antioxidant properties. The research demonstrates that the quality of commercial dry dog foods, particularly regarding oxidative stability and antioxidant properties, is subject to change over time after opening. The chemical composition of the foods was influenced by storage duration, with significant decreases in crude fat and variations in fatty acid profiles.

## 1. Introduction

In Europe, dogs rank as the second most common companion animals, with their population in households estimated at 106 million [1]. They are usually fed commercial dry food, which must be complete and balanced. An important consideration for such foods is their ability to remain safe for consumption over a defined period after opening the package, while maintaining all safe storage rules. To achieve this, manufacturers may incorporate both natural antioxidants and synthetic preservatives, with the choice often influenced by economic factors and the stability of the active substance [2]. Preservatives can have different origins, not only synthetic, and their declaration on labels follows specific regulatory frameworks, such as the FEDIAF Code of Good Labelling Practice and EU Regulation (EC) No 767/2009 and Regulation (EC) No 1831/2003 [3,4,5]. According to these guidelines, certain additives—especially when present in trace amounts that do not affect the product’s characteristics—may not be listed in the detailed ingredient breakdown but only as part of a functional category in the additives section. This can lead to situations where pet caregivers assume that no preservatives are present, although they are included in the formulation.

When a dog develops disturbing symptoms suggesting a suspected food allergy, an elimination diet is typically implemented. Relatively often, caregivers, for convenience and economic reasons, choose an alternative to veterinary food, namely commercial dry foods with a limited content of animal ingredients, which are mainly mono-protein foods. The specificity of these foods includes a composition in which only one animal species was used in their production. Over-the-counter (OTC) limited-ingredient dog foods may serve as dependable substitutes for veterinary therapeutic formulas in diagnosing and managing adverse food reactions (AFR) [6].

What is more, all additives used in pet food must comply with legal requirements and be added in safe amounts, as outlined in European Union Regulation No. 1831 [5]. These additives include antioxidants, which play a critical role in maintaining the oxidative stability of fatty acids (FA), particularly in dry dog foods. Unlike wet foods, dry foods have a low humidity level, typically below 10%, which naturally inhibits the growth of undesirable microorganisms. Consequently, the use of chemical preservatives is less common, with antioxidants being the preferred choice to prevent fat rancidity and protect against oxidative degradation. By preserving the sensory qualities of the food—such as taste, consistency, color, and aroma—antioxidants ensure that the product remains appealing and safe for canine consumption [7].

Fatty acids, particularly unsaturated (UFA), are highly prone to oxidative damage during storage. High levels of UFA in pet foods can lead to rancidity, resulting in significant sensory and nutritional changes [8]. The oxidation of polyunsaturated fatty acids (PUFA) not only affects the sensory characteristics but also reduces nutritional value. This poses particular risks in diets for growing dogs, as the ingestion of oxidized lipids can lead to the formation of harmful compounds that inhibit growth, impair antioxidant status, and weaken immune functions [9,10].

Research indicates that the storage method and composition of pet food significantly impact the safety and nutritional properties of the product. A survey of pet caregivers revealed that most dry food is purchased in large packages, which pets consume for at least four weeks, often storing it in plastic containers in the kitchen, sometimes in conditions that favor elevated temperatures. At the same time, 67.3% of commercial pet food users perceive preservatives as a potential health risk, and many owners store sensitive ingredients, such as fish oil, at room temperature, which can compromise their quality [11]. Usuga et al. [12] showed that dry food can affect the oxidative antioxidant profile of dogs’ blood, but this relationship is not clearly linked to the laboratory-assessed antioxidant content of the food itself. This highlights the need to educate pet caregivers on proper storage and for them to understand the role of technological additives, including antioxidants and preservatives, in product shelf life and safety.

Although the antioxidant properties of individual raw materials and ingredients used in pet food have been studied [13], limited attention has been given to the oxidative stability and antioxidant properties of complete commercial dry dog foods under typical storage conditions [14]. Understanding these factors is crucial, as it has direct implications for the nutritional quality and safety of these products. Therefore, the aim of this research was to analyze the antioxidant properties and oxidative stability of fatty acids in mono-protein extruded dry foods for adult dogs, stored in typical household conditions, as part of a pilot study. To our knowledge, the antioxidant properties of commercial dry dog foods and the evaluation of the changes occurring over an extended storage period are presented for the first time.

The novelty of this study lies in the fact that, although the antioxidant properties of individual pet food ingredients have been investigated previously, none of the latest research has specifically examined the antioxidant properties and oxidative stability of complete, commercially available mono-protein extruded dry dog foods during typical household storage conditions. This pilot study is the first, to the authors’ knowledge, to assess both the antioxidant capacity and the progression of oxidative changes in fatty acids in such products over an extended storage period, providing new insights into their nutritional quality and safety.

## 2. Results

The average dry matter content was 93.59 g/100 g of fresh matter, and the highest content was determined in food F (95.44 g/100 g), which indicates its lowest moisture content at the time of opening the packages. Food D was characterized by the lowest dry matter content (91.42 g/100 g) (*p* ≤ 0.05) (Table 1).

For the crude protein content, the FEDIAF [2] nutritional guidelines provide the minimum recommended level (MRL) of this ingredient as 18.00 or 21.00 g/100 g DM. MRL was met in all analyzed foods, and the average crude protein content was 34.76 g/100 g DM. The highest protein content was found in food B (41.40 g/100 g DM), and the lowest was found in food C 26.67 g/100 g DM. Crude fat is the second ingredient for which FEDIAF (2024b) [2] provides an MRL of 5.50 g/100 g DM. The average fat content in the tested foods was 18.37 g/100 g DM, and its highest level was found in food F (23.37 g/100 g DM) and the lowest in food C (14.17 g/100 g DM) (*p* ≤ 0.05) (Table 2).

**Figure 1:**
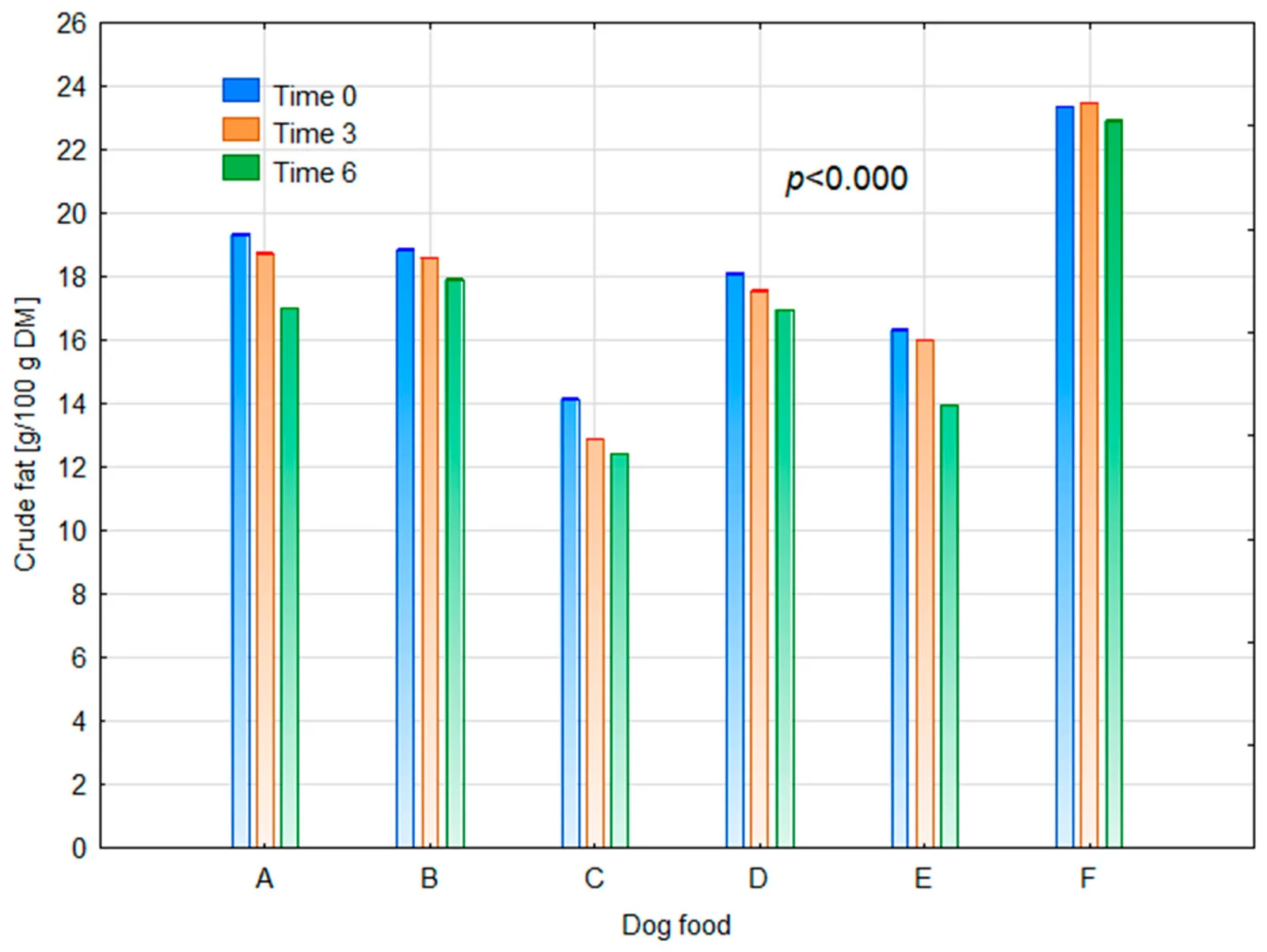
Crude fat content variations in dog foods over time (0, 3, and 6 months after opening); the main animal protein sources declared by the manufacturers were lamb (food A), poultry (food B), insects (food C), fish (foods D and F), and pork (food E); replicate number = 3.

A lower average crude fat content was found in products A-D 3 months after opening the packages, while after 6 months a decrease was observed again. These changes were significant and are shown in Figure 1.

**Figure 2:**
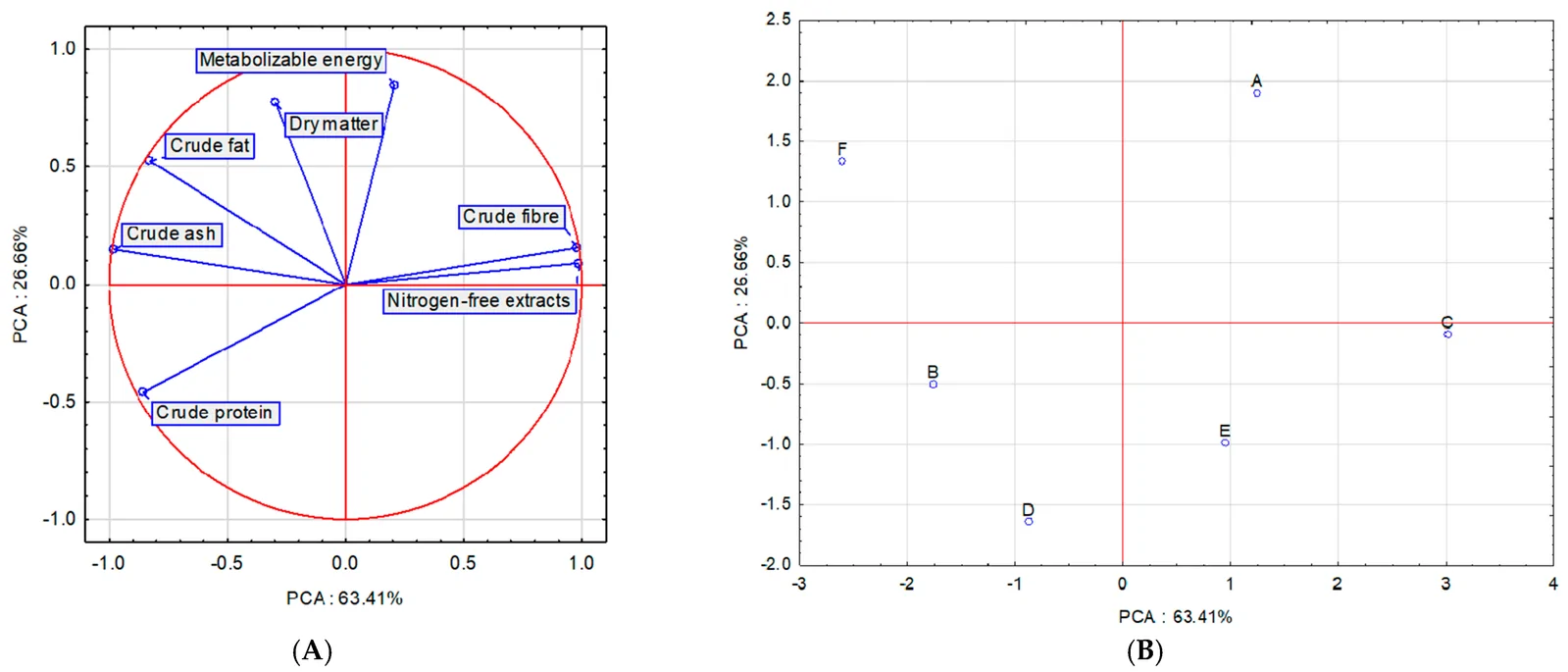
Biplot of the first two principal component axes for the proximate composition and metabolizable energy of dog foods (**A**) and distribution of six dog foods based on these components (**B**); the main animal protein sources declared by the manufacturers were lamb (food A), poultry (food B), insects (food C), fish (foods D and F), and pork (food E); replicate number = 3.

In all foods, the fat content decreased with time that had passed since opening the packages. Food F was the only one in which the fat content increased after 3 months, but it decreased after 6 months. The average content of crude ash in the analyzed foods was 7.58 g/100 g DM. Its highest level was found in food F (9.45 g/100 g DM), and the lowest was found in food C (6.02 g/100 g DM). The highest level of crude fiber was found in food C (6.09 g/100 g DM), and the lowest was found in food F (3.78 g/100 g DM). The average content of this ingredient in the tested foods was 4.84 g/100 g DM. The average content of nitrogen-free extracts was 34.45 g/100 g DM; the highest was found in food C (47.04 g/100 g DM), and the lowest was found in food F (26.93 g/100 g DM). PCA analysis showed that the first two components account for 90.07% of the total variance (Figure 2A). It is clear from this analysis that the proximate composition profiles of foods B and D are very similar, and these products are located in the third quadrant of the coordinate system (Figure 2B). Their common feature is a high content of crude protein, with a low content of crude fiber and nitrogen-free extracts. In terms of ingredients, both foods contain sunflower oil as a source of vegetable fat and peas as the main plant component. The coefficient coordinates of the remaining foods (A, C, F) place them in the remaining quadrants of the periodic table (Figure 2B), which clearly indicates their different proximate composition profiles.

In the case of the saturated fatty acids (SFA), their average content in the tested foods changed significantly during storage. Their average content at the time of opening the packages was 36.57% of the sum of fatty acids (Table 3). After 3 months, the content decreased to 32.83% of the sum of FA, and after 6 months it increased in relation to the amount in the third month and amounted to 33.10% of the sum of FA (*p* ≤ 0.05).

**Figure 3:**
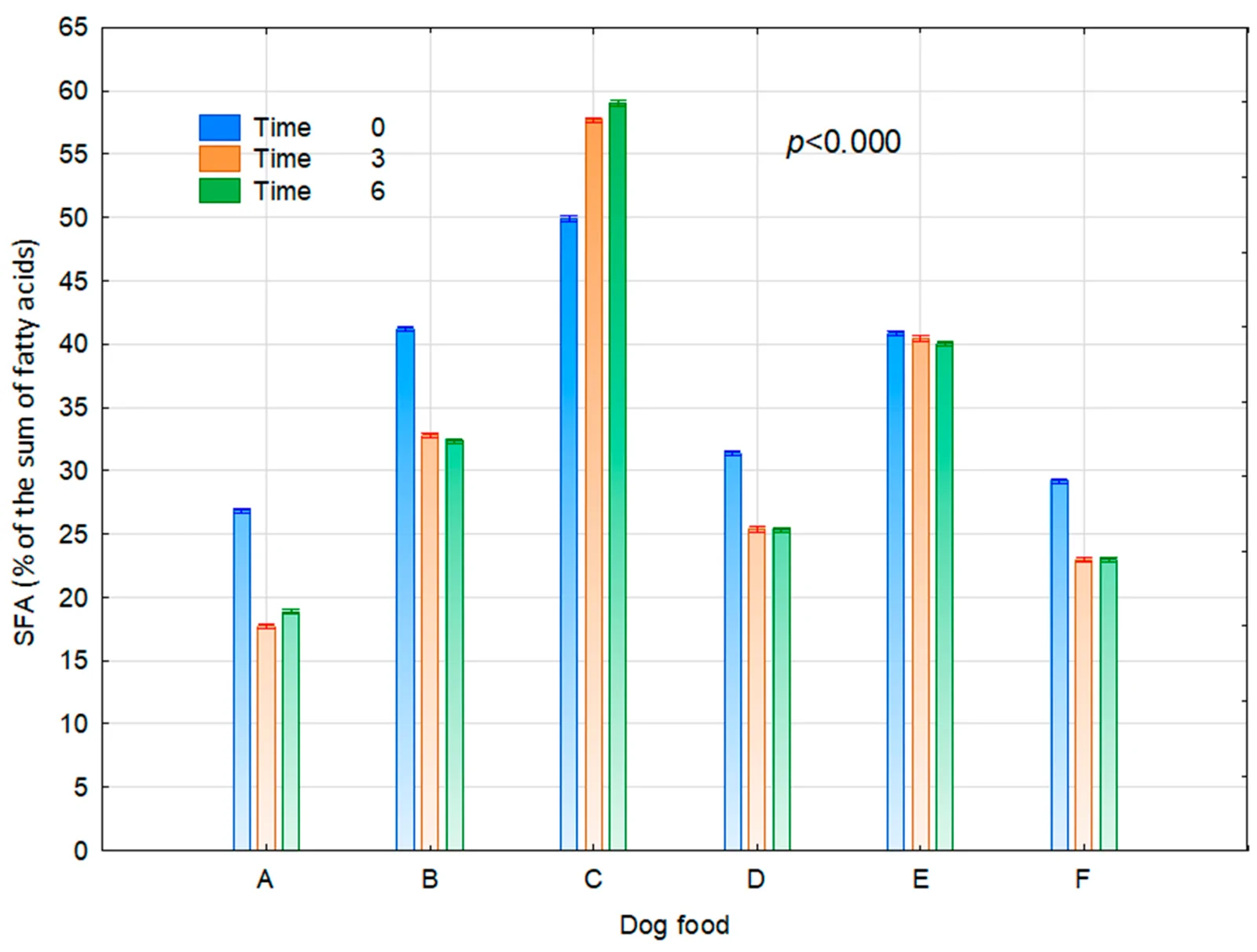
Saturated fatty acid (SFA) content variations in dog foods over time (0, 3, and 6 months after opening); the main animal protein sources declared by the manufacturers were lamb (food A), poultry (food B), insects (food C), fish (foods D and F), and pork (food E); replicate number = 3.

The fatty acid present in the largest amount was C16:0. Its average content at the time of opening the packages was 20.72% FA, which decreased to 16.75% FA after 3 months and decreased again after 6 months to 16.57% FA. The next highest content was C18:0 fatty acid, the content of which at the time of opening the packages was 7.61% FA. After 3 months, this amount decreased to 6.13% FA, and after 6 months, it decreased to 6.03% FA. Food C had the highest SFA content, as shown in Table 3. This was also the only one in which the SFA content increased over time (Figure 3). The observed increase in saturated fatty acids in dog food C during storage may result from the oxidative degradation of unsaturated fatty acids, leading to a relative increase in SFA content. This could be due to a higher initial PUFA content combined with insufficient antioxidant protection. Alternatively, it may reflect differences in the lipid source or matrix stability specific to this formulation.

**Figure 4:**
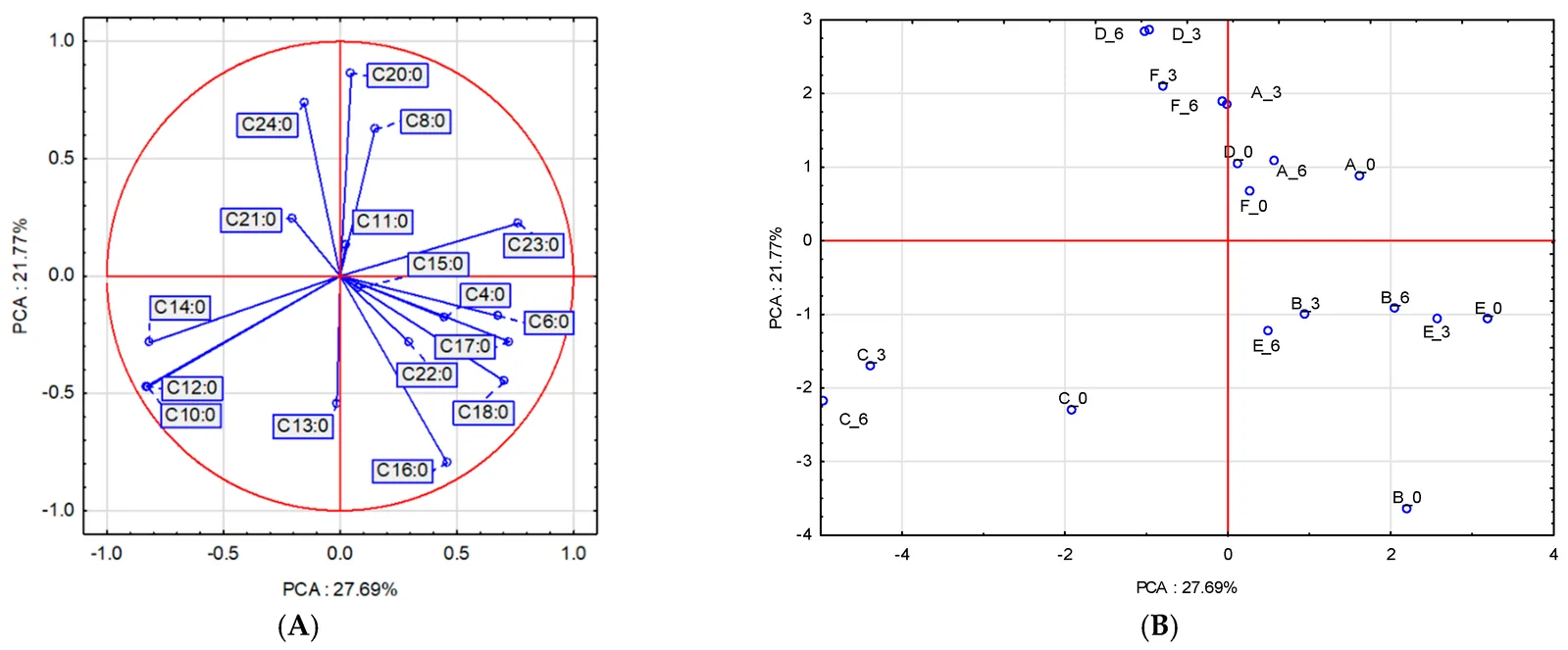
Biplot of the first two principal component axes for the saturated fatty acid (SFA) content of dog foods (**A**) and the distribution of six dog foods based on these components (**B**); the main animal protein sources declared by the manufacturers were lamb (food A), poultry (food B), insects (food C), fish (foods D and F), and pork (food E); replicate number = 3.

In the remaining foods, after 3 months, the SFA content decreased significantly, and after 6 months, it decreased again, although not to such an extent as in the first three months. PCA analysis showed that the first two components account for 49.46% of the total variance (Figure 4A). It is clear from this analysis that the SFA content profiles of foods B and E are very similar, and these products are located in the second quadrant of the coordinate system (Figure 4B). Similarly, in the case of foods A, D, and F, the SFA contents in these foods were similar; therefore, they are located in quadrants I and II of the coordinate system. The coefficient coordinates of food C place it in the third quarter (Figure 4B), which clearly indicates its different SFA content profile compared to the other foods.

Among the unsaturated fatty acids, C18:1 (n-9 cis) fatty acid was present in the largest amount. After opening the packages, its average content in all foods was 40.19% FA; after 3 months, it decreased to 39.84% FA, and after 6 months, it decreased again to 39.28% FA. Another fatty acid present in the greatest number was C18:2 (n-6 cis). After opening the packages, the average content of this acid was 13.20% FA (*p* ≤ 0.05) (Table 4). After 3 months after opening, the content increased to 15.42% FA, while after 6 months, the content of this acid slightly decreased to 15.32% FA. From a nutritional point of view, C20:5 (n-3) and C22:6 (n-3) fatty acids are particularly important in the diet of companion animals. In the case of the first fatty acid, its average content remained unchanged throughout the entire period of storage of the foods and amounted to 0.03% FA. In the case of C22:6 (n-3) acid, its average content at the time of opening the packages was 0.50% FA, then it increased to 0.88, and after 6 months, it increased to 0.97% FA. The tested foods also contained unidentified fatty acids. When the packages were opened, their average content was 0.32% FA (Table 4). After 3 months, it increased to 0.41, and after 6 months, it dropped below the content found at the beginning to 0.28% FA (*p* ≤ 0.05).

**Figure 5:**
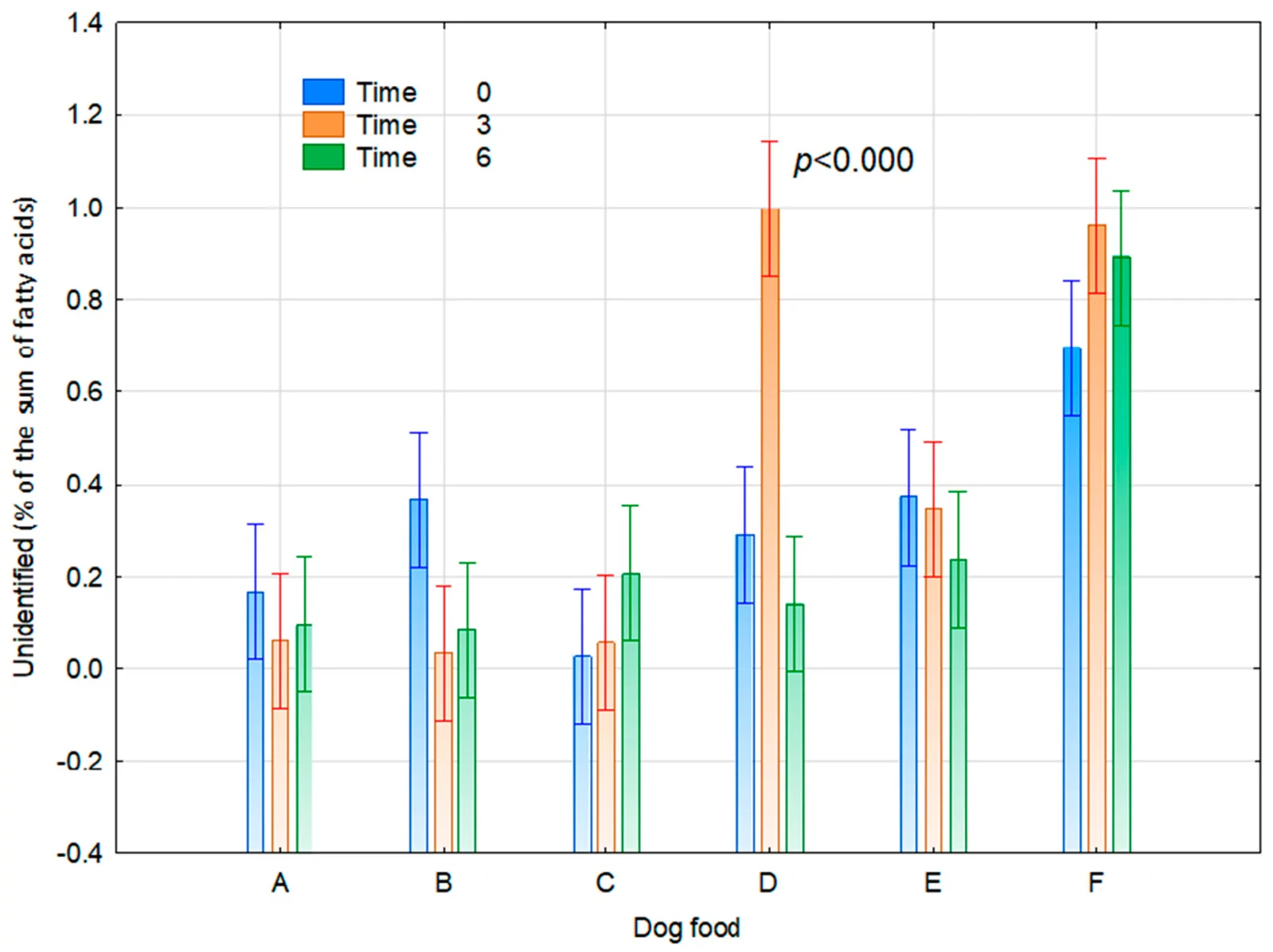
Unidentified fatty acid content variations in dog foods over time (0, 3, and 6 months after opening); the main animal protein sources declared by the manufacturers were lamb (food A), poultry (food B), insects (food C), fish (foods D and F), and pork (food E); replicate number = 3.

**Figure 6:**
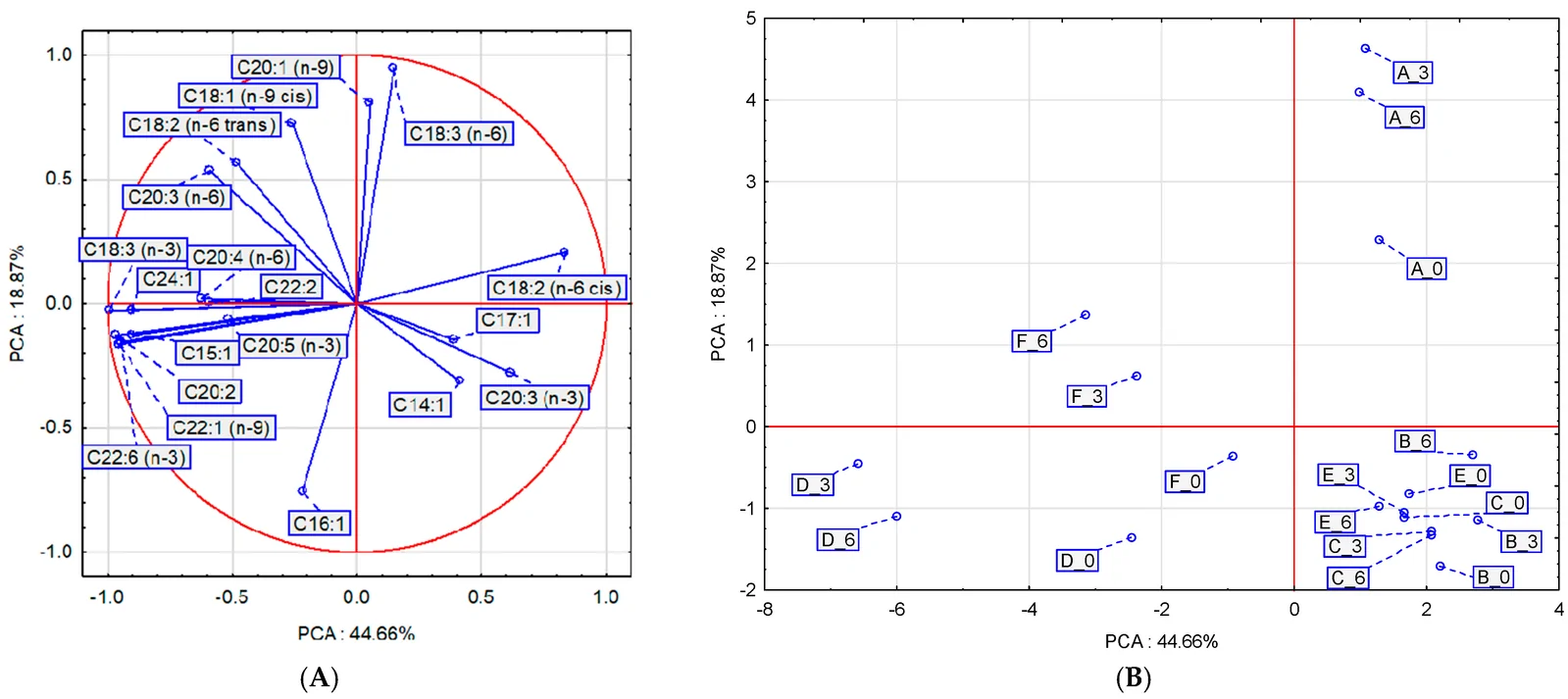
Biplot of the first two principal component axes for the unsaturated fatty acid (UFA) content of dog foods (**A**) and the distribution of six dog foods based on these components (**B**); the main animal protein sources declared by the manufacturers were lamb (food A), poultry (food B), insects (food C), fish (foods D and F), and pork (food E); replicate number = 3.

Unidentified fatty acid content in individual foods is illustrated in Figure 5. The content of these acids increased to the greatest extent in food D after 3 months of storage, then, it more than doubled, reaching a level lower than when the packages were opened. In the case of unsaturated fatty acids, PCA analysis showed that the first two components account for 63.53% of the total variance (Figure 6A). From this analysis, it is clear that the UFA content profiles of foods B, C, and E are very similar, and these products are located in the second quadrant of the coordinate system (Figure 6B). Similarly, in the case of foods D and F, the UFA contents in these foods were similar; therefore, they are located in quadrants II and III of the coordinate system. The coefficient coordinates of food A place it in the first quarter (Figure 6B), which clearly indicates its different UFA content profile compared to the other foods.

In the case of MUFA and PUFA, changes in the content of these groups were not significant over time (Table 5). Taking into account n-3 and n-6 acids, significant changes were observed during the storage period of food. At the time of opening the packages, the content of n-3 fatty acids was 1.99% FA. After 3 months, an increase to 3.02% FA was observed, and after 6 months, it reached 3.12% FA. The richest in terms of n-3 fatty acid content was food D (Table 5).

**Figure 7:**
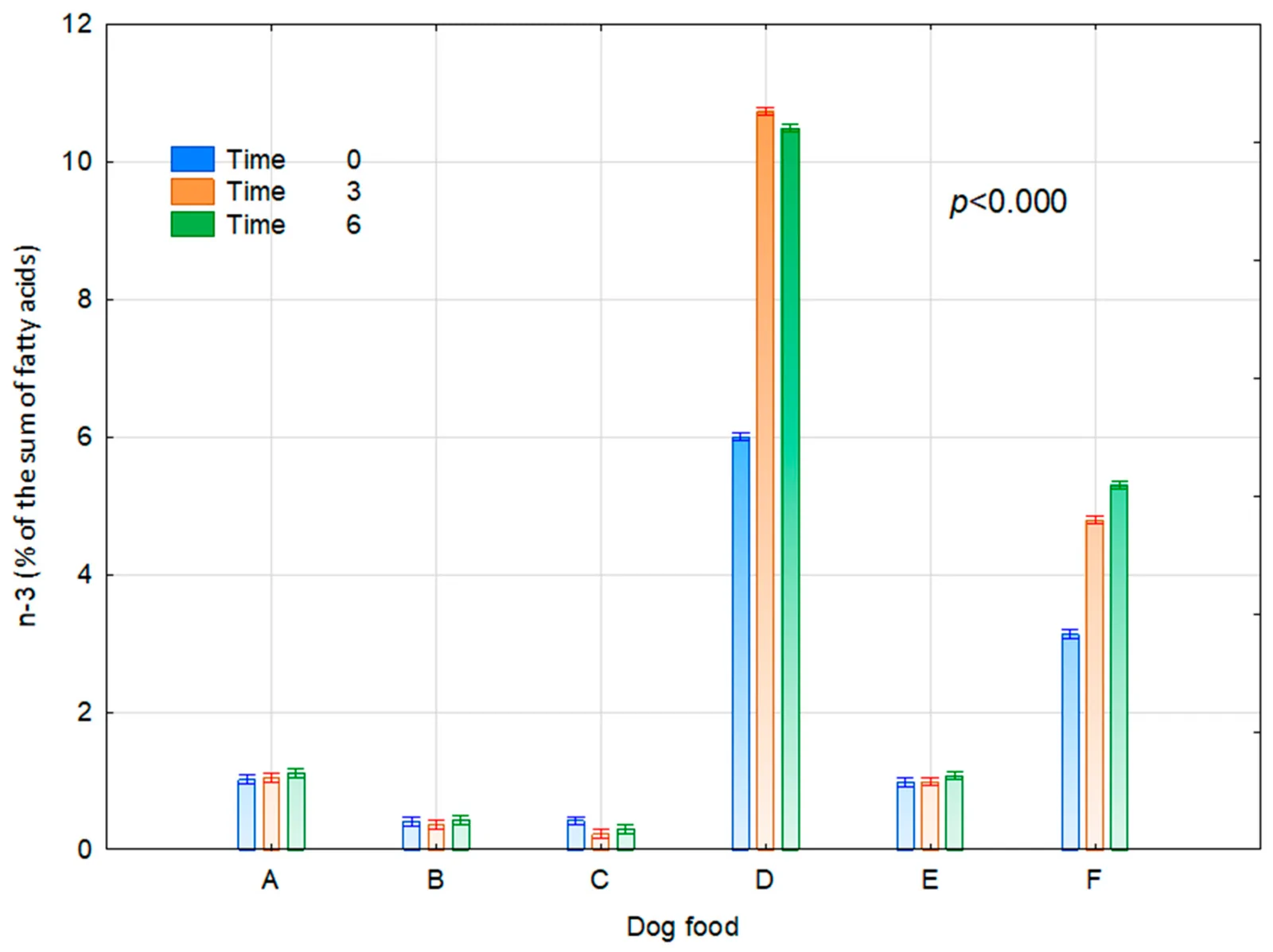
The n-3 fatty acid content variations in dog foods over time (0, 3, and 6 months after opening); the main animal protein sources declared by the manufacturers were lamb (food A), poultry (food B), insects (food C), fish (foods D and F), and pork (food E); replicate number = 3.

**Figure 8:**
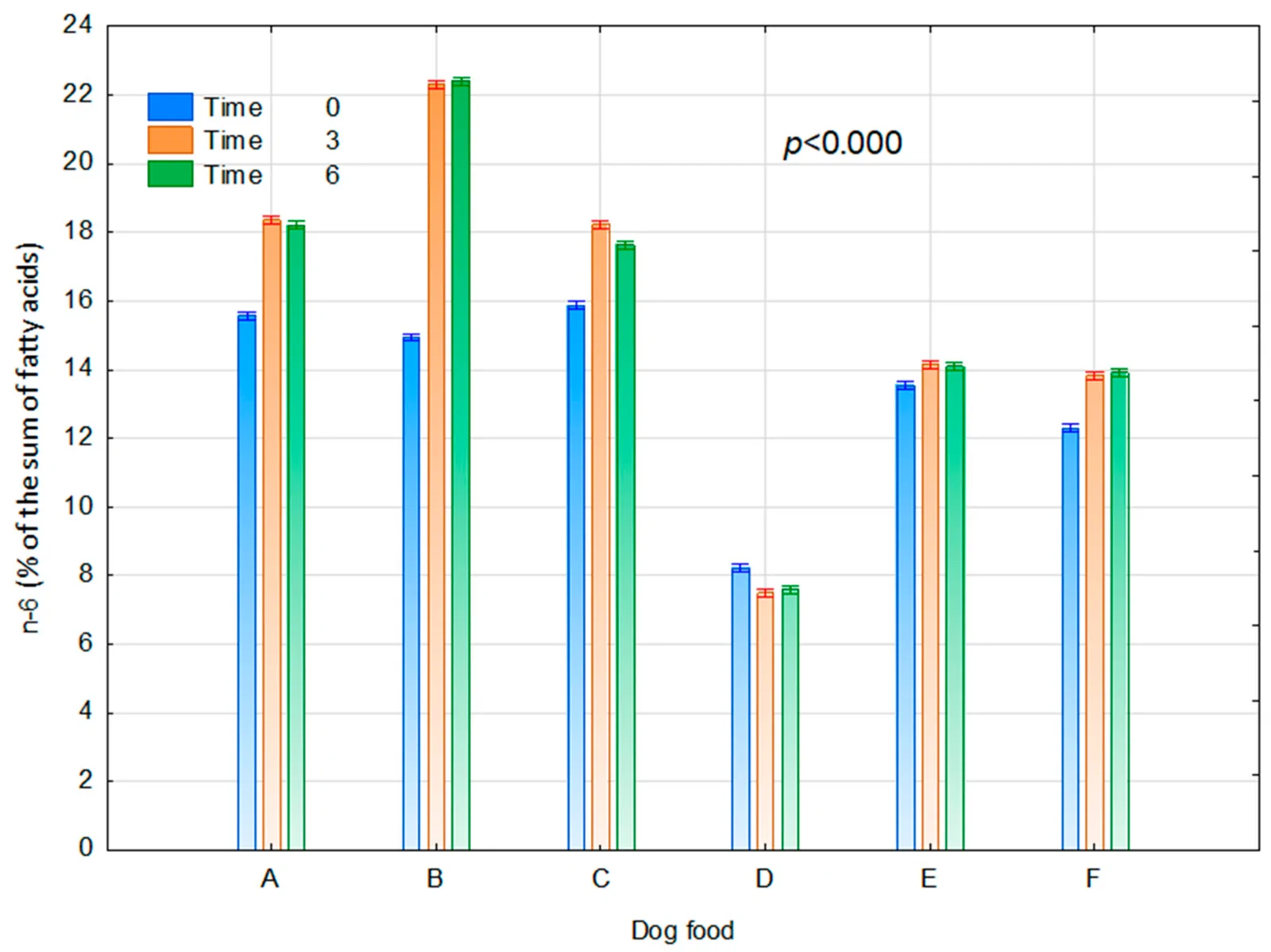
The n-6 fatty acid content variations in dog foods over time (0, 3, and 6 months after opening); the main animal protein sources declared by the manufacturers were lamb (food A), poultry (food B), insects (food C), fish (foods D and F), and pork (food E); replicate number = 3.

A similarity between changes in the content of n-3 acids (over time) in D and F foods was observed (Figure 7). Particular attention is paid to the D and E foods, in which no increases in the content of n-6 acids over time were observed (Figure 8). The average in the tested foods after opening the packages was 13.41% FA, then after 3 months it increased to 15.73% FA, and after 6 months it dropped to 15.65% FA. Therefore, the n-6/n-3 fatty acid ratio changed over time. After opening the packages, this ratio was 18.11; after 3 months, it increased significantly to 29.53, and then after 6 months, it dropped to 24.01. As shown in Table 5, n-6/n-3 acid ratios were the highest in foods B and C, while food D had the lowest ratios.

**Figure 9:**
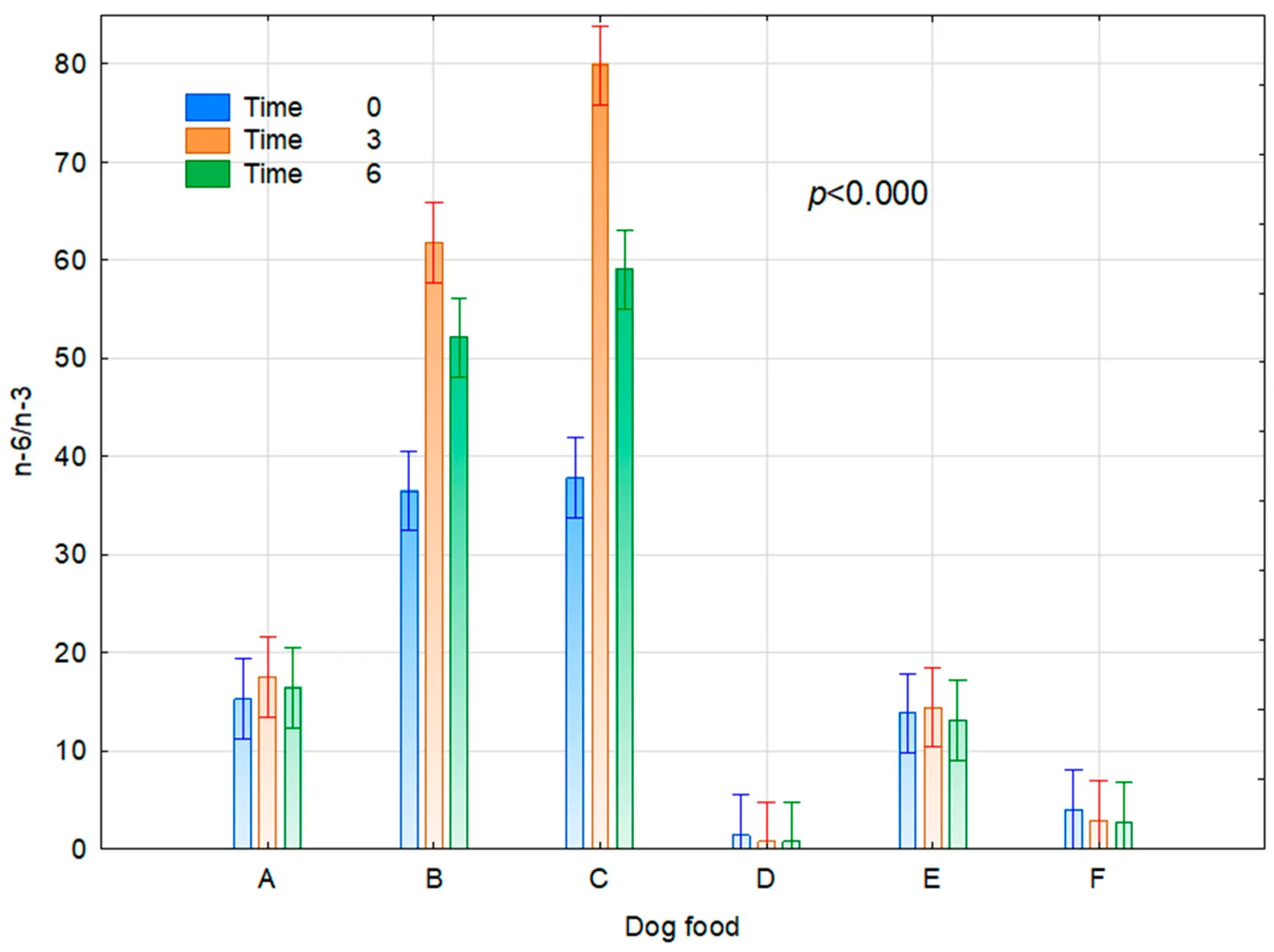
The n-6/n-3 fatty acid ratio variations in dog foods over time (0, 3, and 6 months after opening); the main animal protein sources declared by the manufacturers were lamb (food A), poultry (food B), insects (food C), fish (foods D and F), and pork (food E); replicate number = 3.

**Figure 10:**
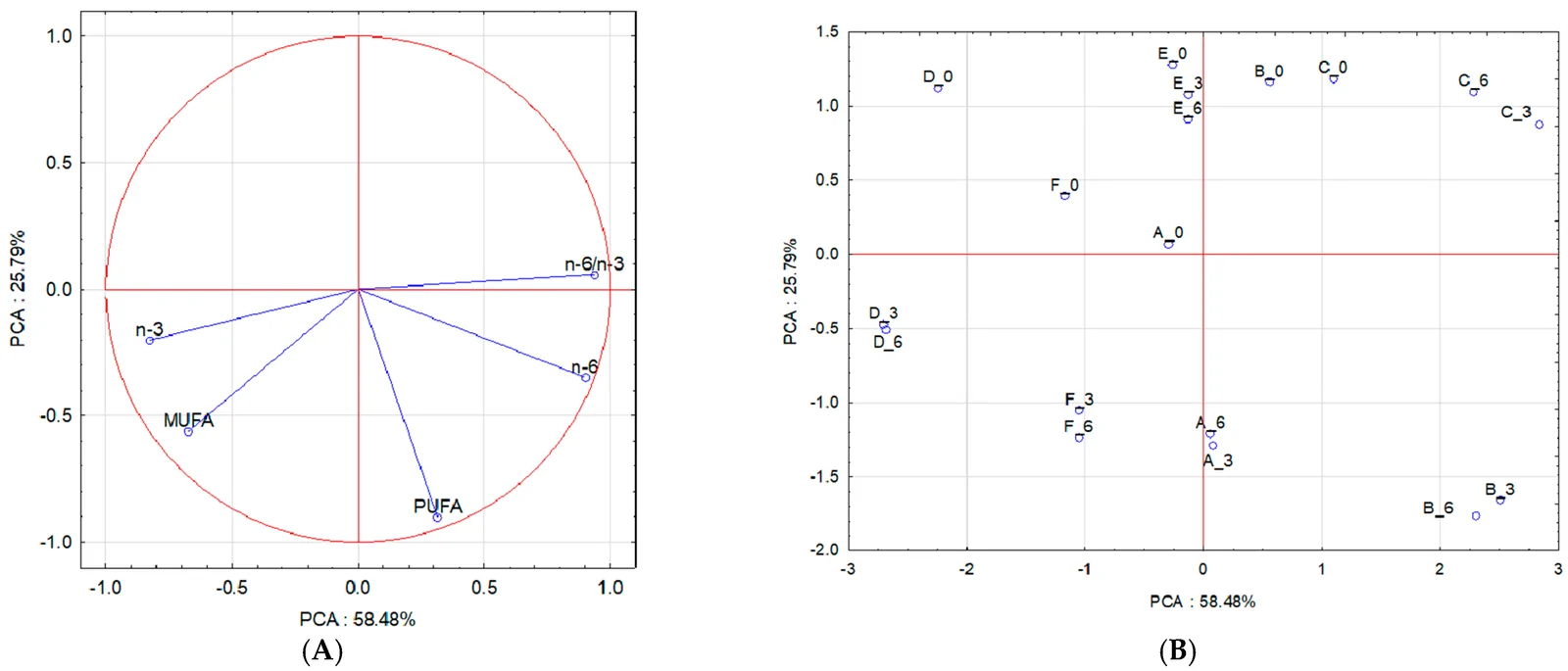
Biplot of first two principal component axes for groups of fatty acids in dog foods (**A**) and distribution of six dog foods based on these components (**B**); the main animal protein sources declared by the manufacturers were lamb (food A), poultry (food B), insects (food C), fish (foods D and F), and pork (food E); replicate number = 3.

Particular attention is paid to foods B and C, in which the ratio of n-6/n-3 acids was high compared to other foods at the time of opening the packages, then it increased significantly after 3 months, and then it decreased after 6 months (Figure 9). In the case of individual groups of fatty acids, PCA analysis showed that the first two components account for 84.27% of the total variance (Figure 10A). The PCA analysis shows that the behaviors of foods E and C were separate, as in their cases the storage time after opening does not modify the analyzed indicators (Figure 10B).

**Figure 11:**
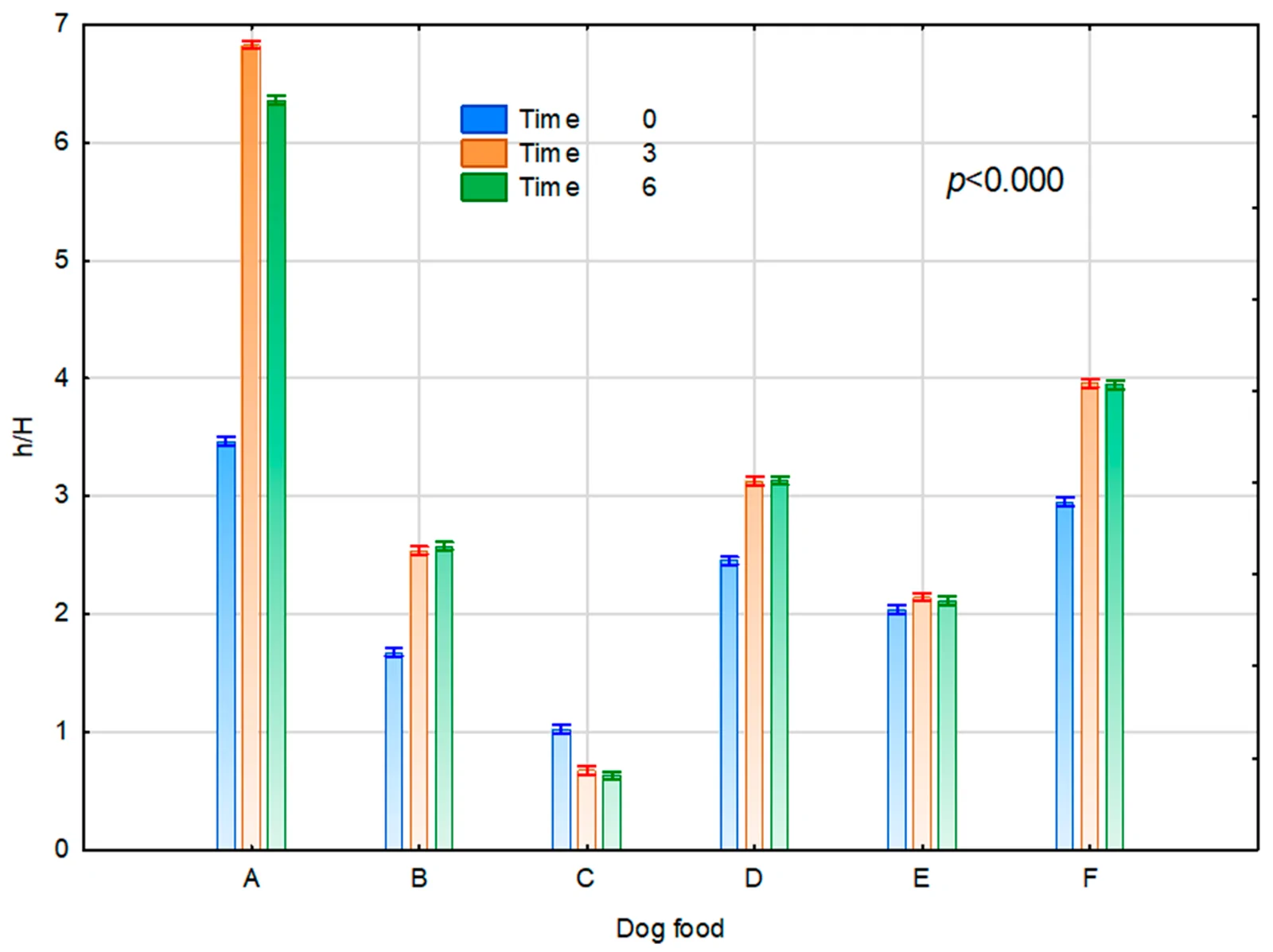
The h/H indicator variations in dog foods over time (0, 3, and 6 months after opening); the main animal protein sources declared by the manufacturers were lamb (food A), poultry (food B), insects (food C), fish (foods D and F), and pork (food E); replicate number = 3.

**Figure 12:**
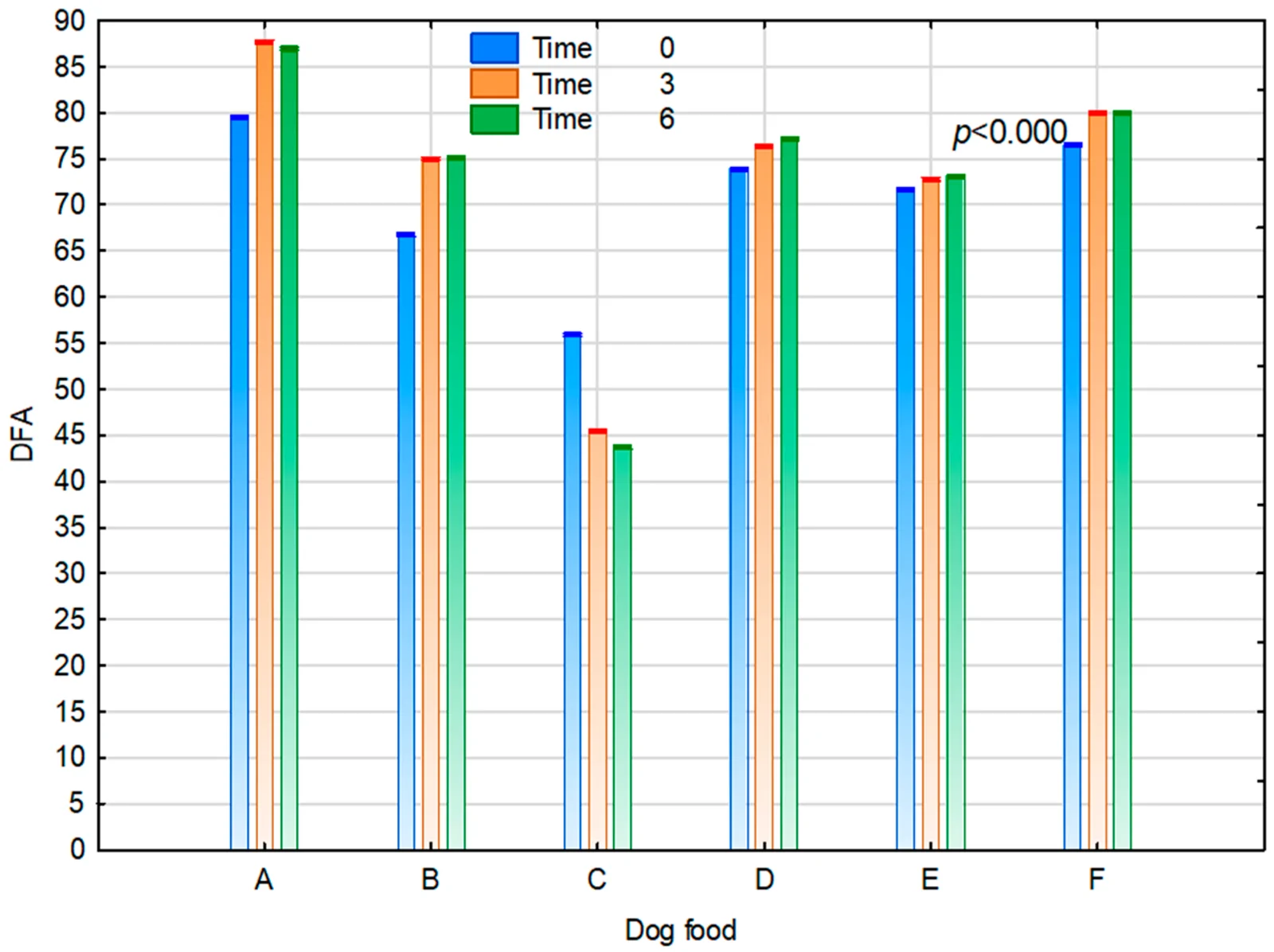
The hypocholesterolemic fatty acid (DFA) variations in dog foods over time (0, 3, and 6 months after opening); the main animal protein sources declared by the manufacturers were lamb (food A), poultry (food B), insects (food C), fish (foods D and F), and pork (food E); replicate number = 3.

**Figure 13:**
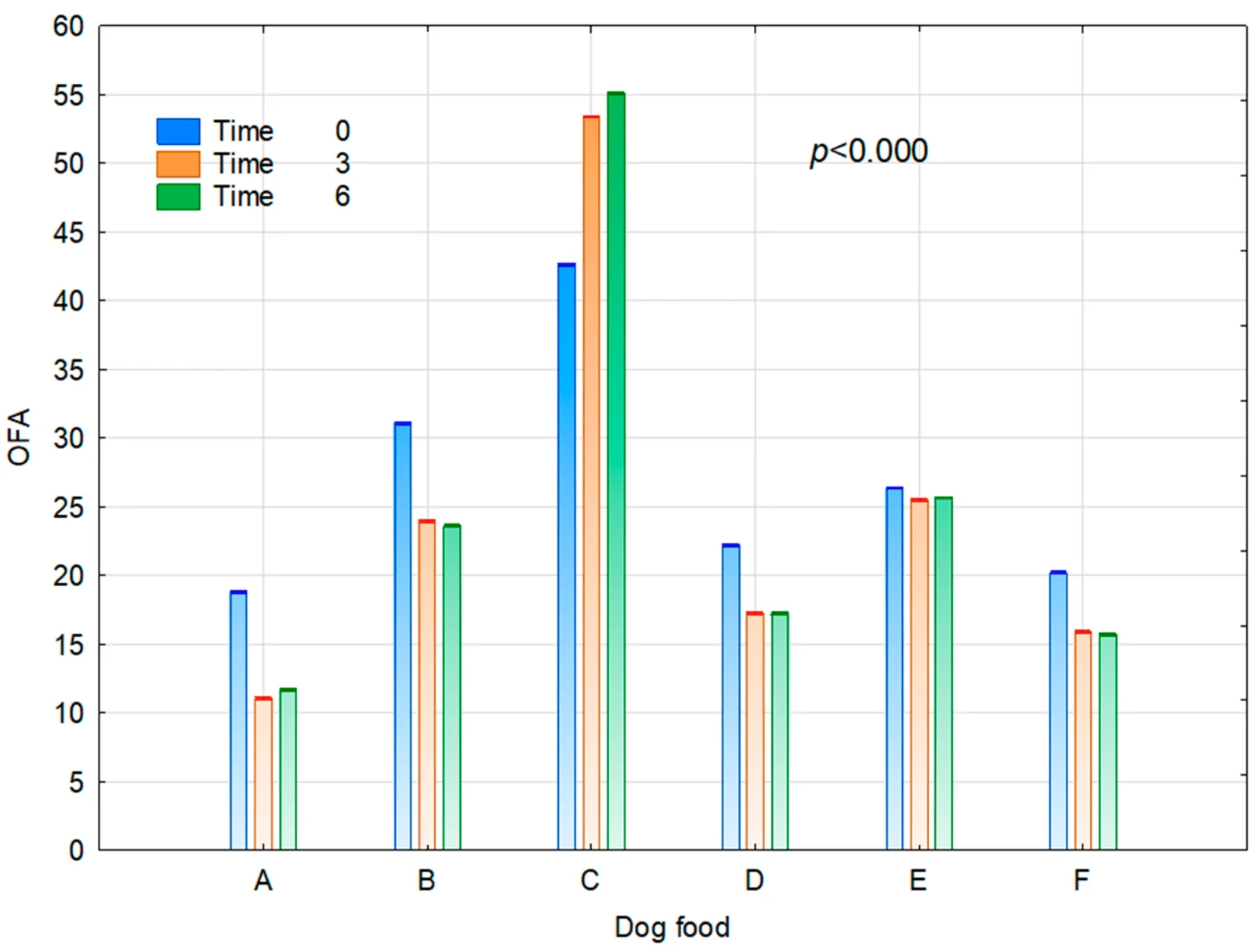
The hypercholesterolemic fatty acid (OFA) variations in dog foods over time (0, 3, and 6 months after opening); the main animal protein sources declared by the manufacturers were lamb (food A), poultry (food B), insects (food C), fish (foods D and F), and pork (food E); replicate number = 3.

**Figure 14:**
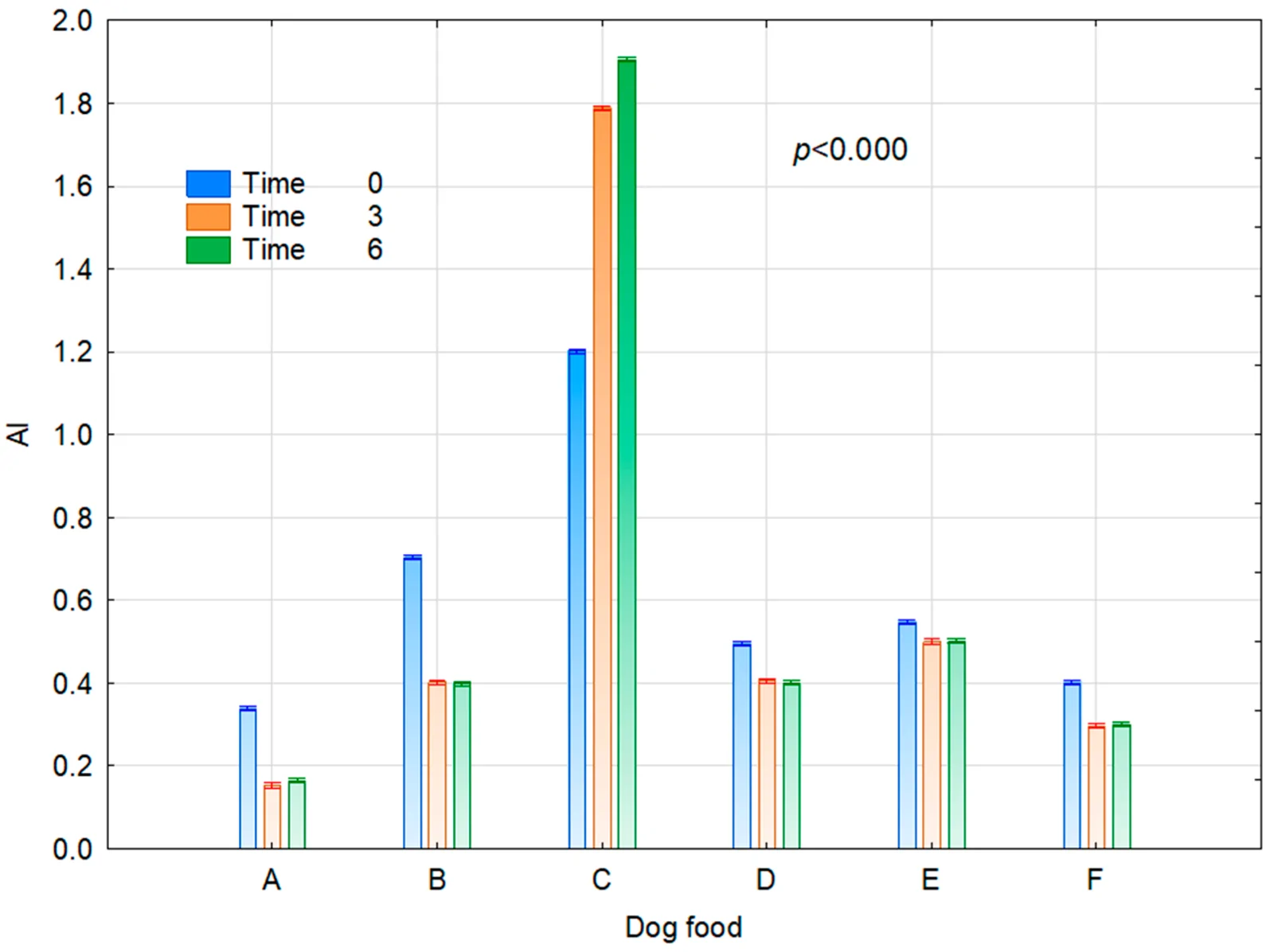
The atherogenic index (AI) variations in dog foods over time (0, 3, and 6 months after opening); the main animal protein sources declared by the manufacturers were lamb (food A), poultry (food B), insects (food C), fish (foods D and F), and pork (food E); replicate number = 3.

**Figure 15:**
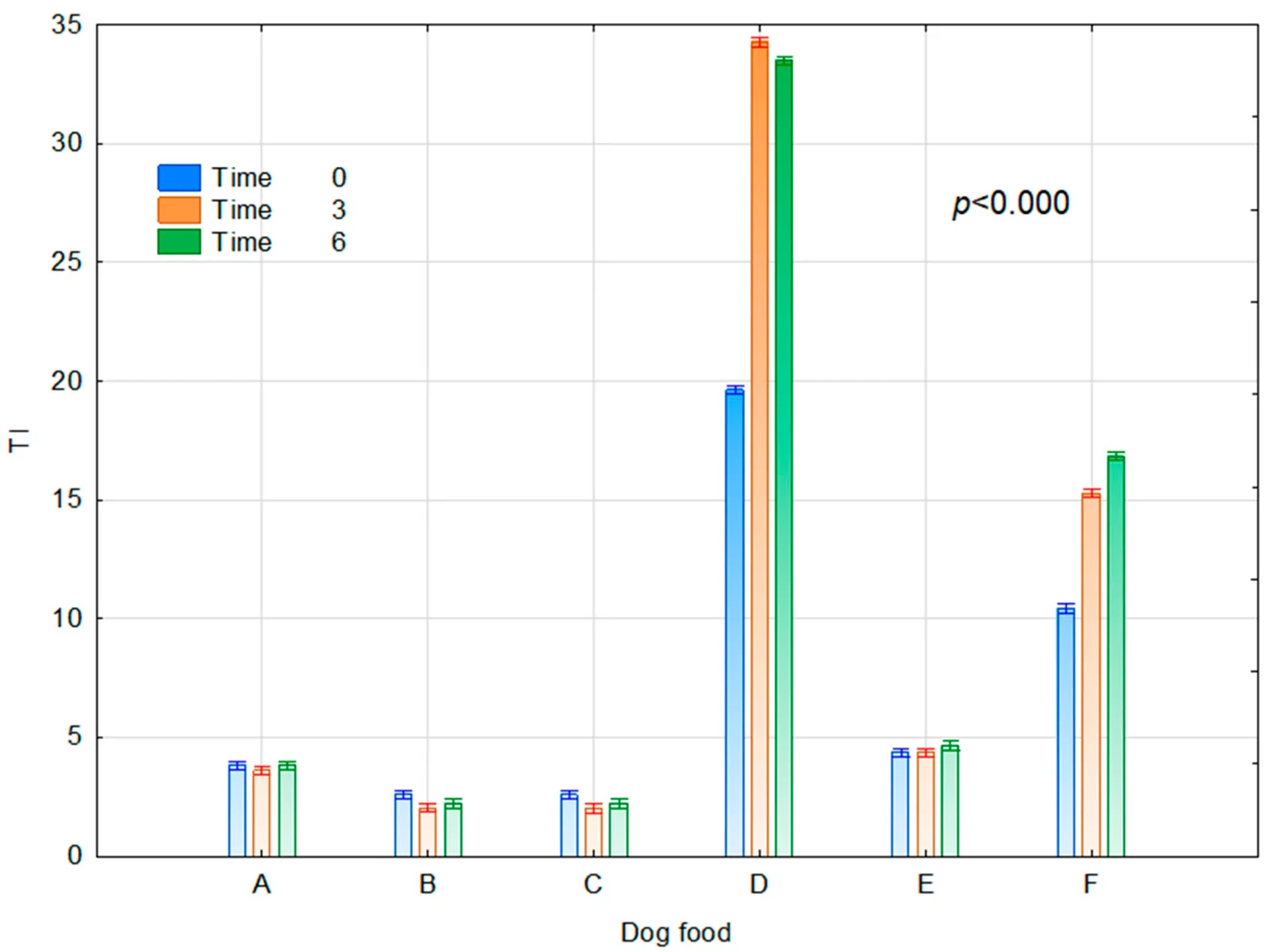
The thrombogenic index (TI) variations in dog foods over time (0, 3, and 6 months after opening); the main animal protein sources declared by the manufacturers were lamb (food A), poultry (food B), insects (food C), fish (foods D and F), and pork (food E); replicate number = 3.

**Figure 16:**
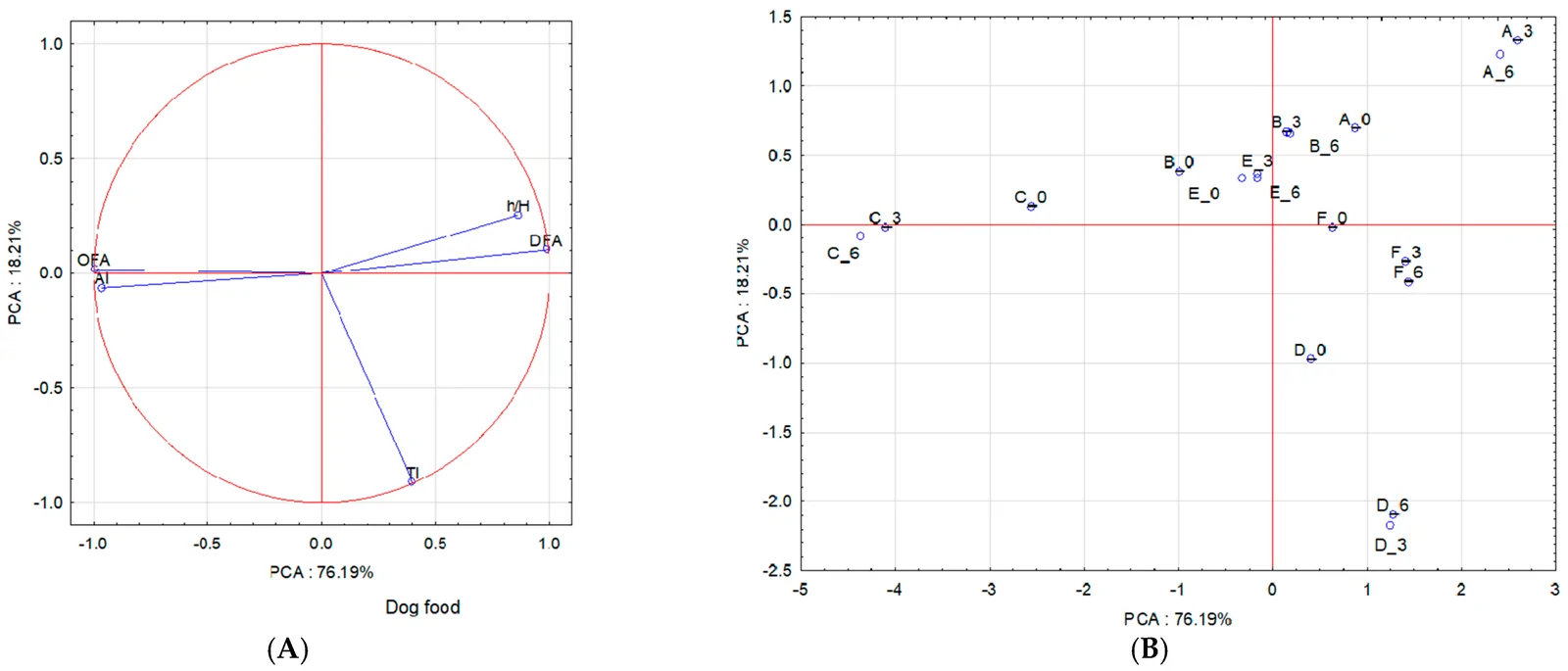
Biplot of the first two principal component axes for lipid quality indicators of dog foods (**A**) and the distribution of six dog foods based on these components (**B**); the main animal protein sources declared by the manufacturers were lamb (food A), poultry (food B), insects (food C), fish (foods D and F), and pork (food E); replicate number = 3.

The quality of the fatty acids of pet foods and other products is determined not only by the content of individual groups and individual fatty acids but also by fatty acid quality indicators. Therefore, this study analyzed the following five indicators: h/H—hypocholesterolemic/hypercholesterolemic acids, DFA—hypocholesterolemic acids, OFA—hypercholesterolemic acids, AI—atherogenic index, and TI—thrombogenic index. According to the data presented in Table 5, the vast majority of fatty acid quality indicators achieved different values depending on the time during which their value was analyzed. Individual foods differed significantly in terms of the h/H ratio. Special attention should be paid to foods C and E. In the first case, the analyzed parameter decreased over time, while in the second, no changes were observed. The remaining foods behave differently (Figure 11). A similar situation was observed in terms of the DFA, as shown in Figure 12. In the case of OFA, the same distinct behavior of C and E foods can be seen (Figure 13). The highest AI was found in food C, and the lowest was in food A (Table 5). Figure 14 clearly shows an increase in the AI parameter value over time only for food C. The remaining foods decrease the value of this parameter over time, i.e., after 3 and 6 months after opening. In the case of TI, the highest value was obtained for food D (Table 5). What is important is the different behavior of D and F foods (Figure 15). In the case of lipid fraction quality indicators, PCA analysis showed that the first two components account for 94.40% of the total variance (Figure 16A,B). This analysis clearly shows the separation of foods C, D, and A from the others forming the central group.

The Pearson correlation coefficient was also analyzed (Table 6). Full correlation was found between the value of n-3 fatty acids and the TI. Regression equations and coefficients of determination of observed and statistically documented dependencies of the analyzed parameters are presented in Table 7.

The polyphenol content changed over time after opening the packages. After three months, its average content decreased significantly and amounted to 361.00 mg CAE/100 g DM, while 6 months after opening the packages, the average polyphenol content in the tested foods increased to 519.40 mg CAE/100 g DM (Table 8).

**Figure 17:**
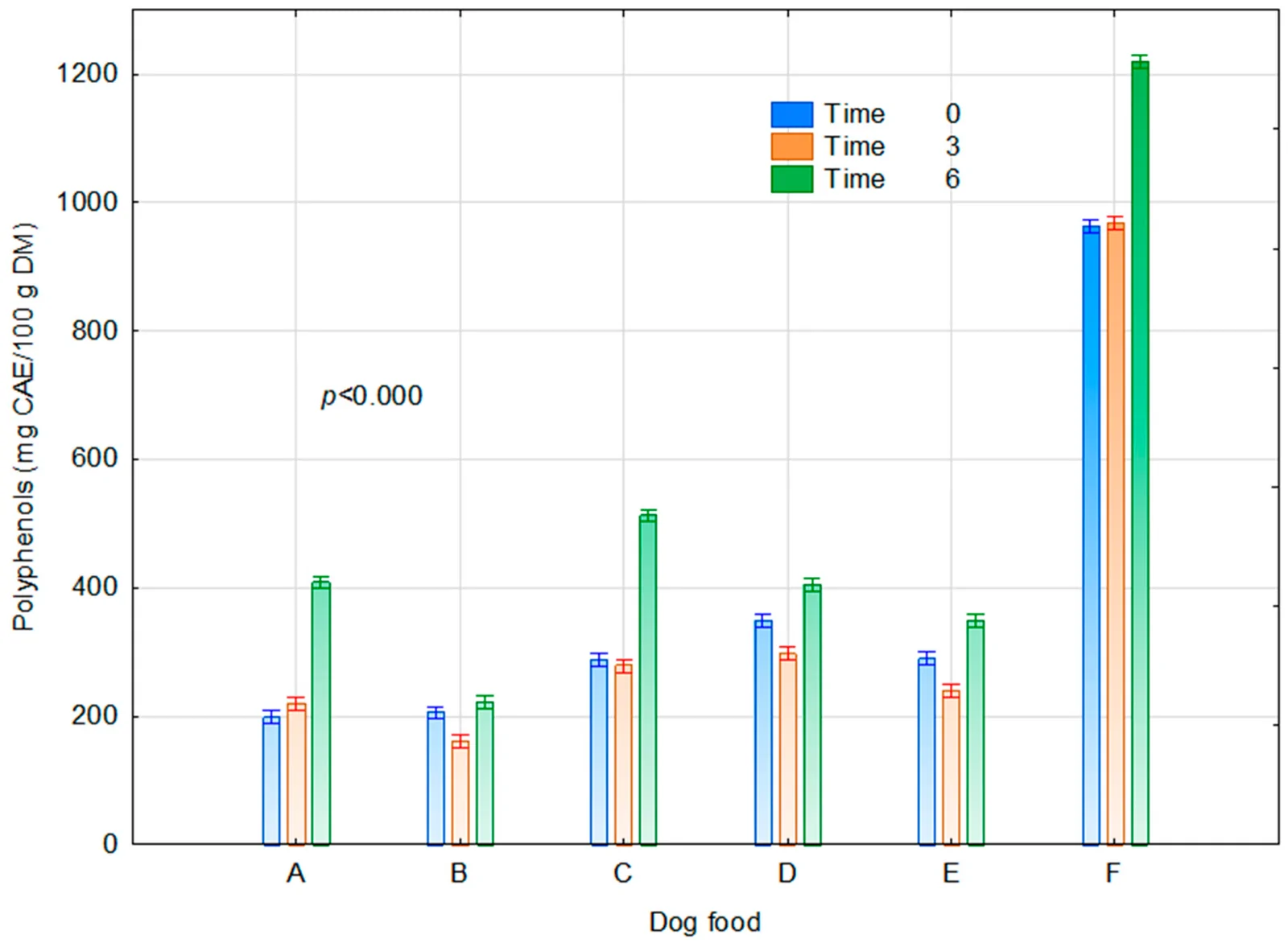
The polyphenol content (mg CAE/100 g DM) variations in dog foods over time (0, 3, and 6 months after opening); the main animal protein sources declared by the manufacturers were lamb (food A), poultry (food B), insects (food C), fish (foods D and F), and pork (food E); replicate number = 3.

**Figure 18:**
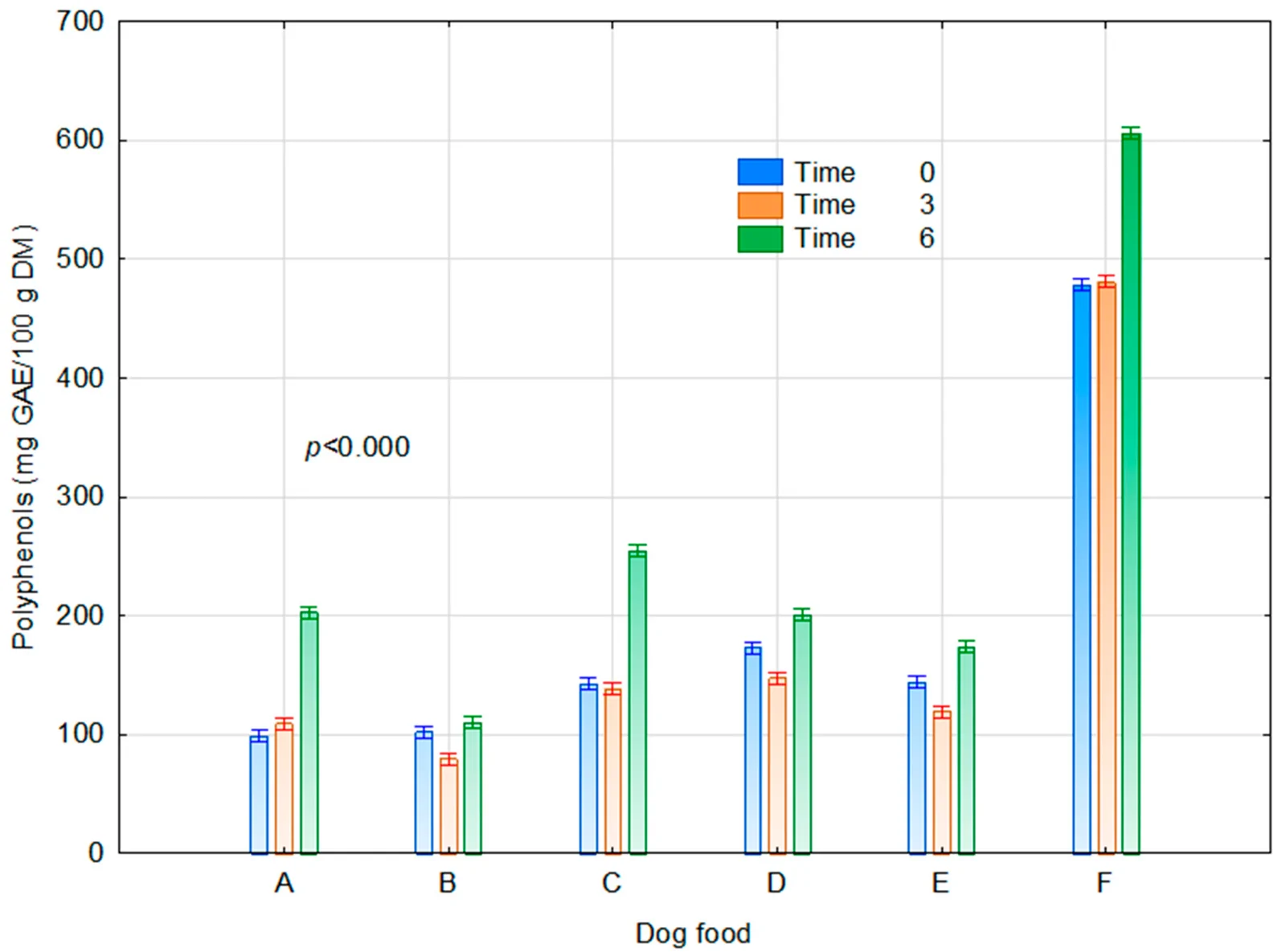
The polyphenol content (mg GAE/100 g DM) variations in dog foods over time (0, 3, and 6 months after opening); the main animal protein sources declared by the manufacturers were lamb (food A), poultry (food B), insects (food C), fish (foods D and F), and pork (food E); replicate number = 3.

The stability of the polyphenol content in foods B, D, and E can be seen during storage of the packages after opening, as shown in Figure 17 and Figure 18. The average of DPPH for all foods decreased to 5.78 µM Trolox/1 g DM 3 months after opening the packages, while after 6 months, it increased, reaching the level of 7.07 µM Trolox/1 g DM. In the case of FRAP, its average after 3 months dropped significantly to 18.48 µM Trolox/1 g DM, and after 6 months, it increased to 23.59 µM Trolox/1 g DM. In the case of ABTS, a similar relationship was observed; its average after 3 months dropped to 14.09 µM Trolox/1 g DM, and after 6 months, it increased to 18.74 µM Trolox/1 g DM. As for RSA ABTS, its average increased after 3 months to 53.51%, and after 6 months, it increased again, reaching the level of 60.73% (Table 8).

**Figure 19:**
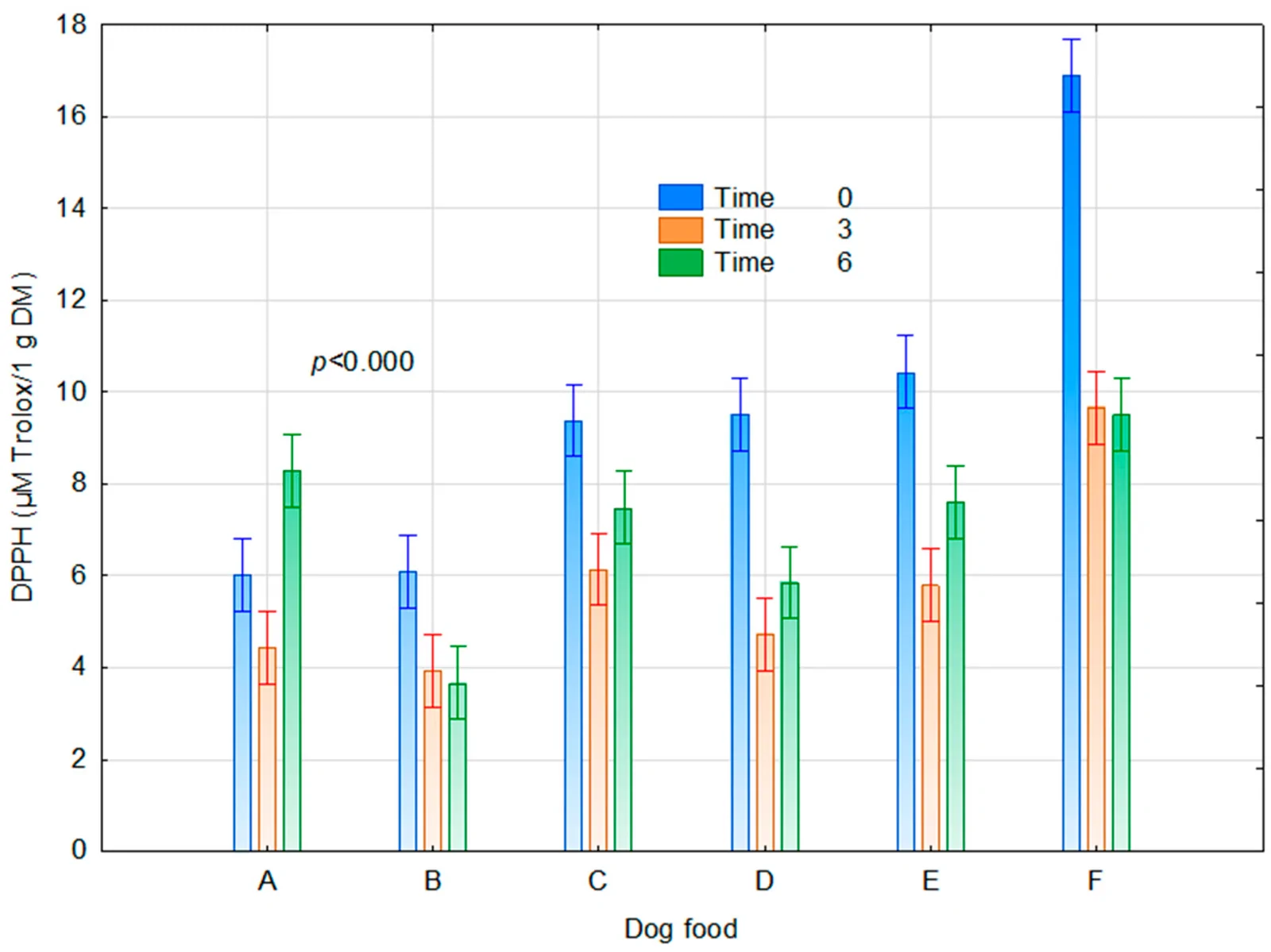
The DPPH (µM Trolox/1 g DM) variations in dog foods over time (0, 3, and 6 months after opening); the main animal protein sources declared by the manufacturers were lamb (food A), poultry (food B), insects (food C), fish (foods D and F), and pork (food E); replicate number = 3.

**Figure 20:**
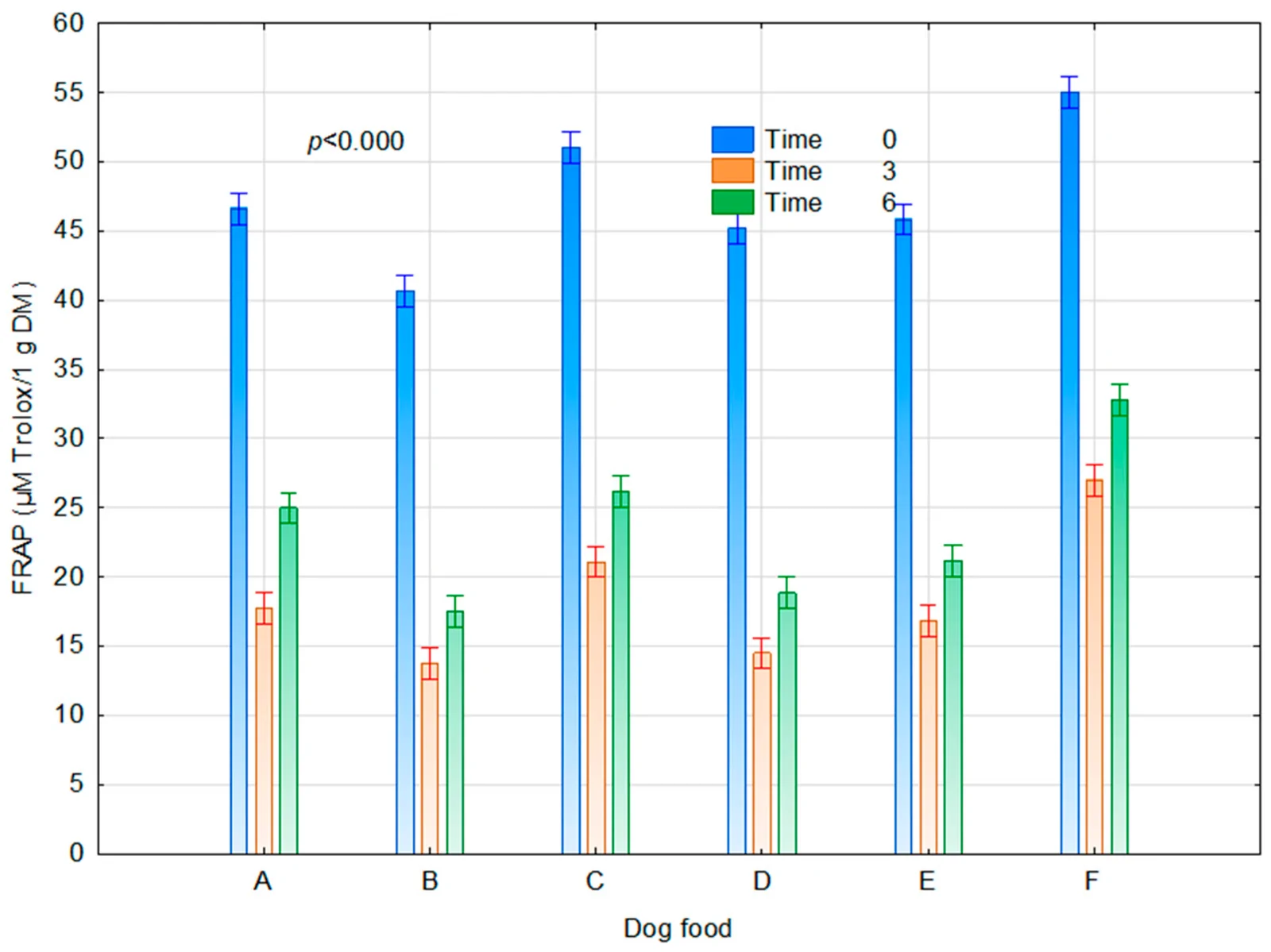
The FRAP (µM Trolox/1 g DM) variations in dog foods over time (0, 3, and 6 months after opening); the main animal protein sources declared by the manufacturers were lamb (food A), poultry (food B), insects (food C), fish (foods D and F), and pork (food E); replicate number = 3.

**Figure 21:**
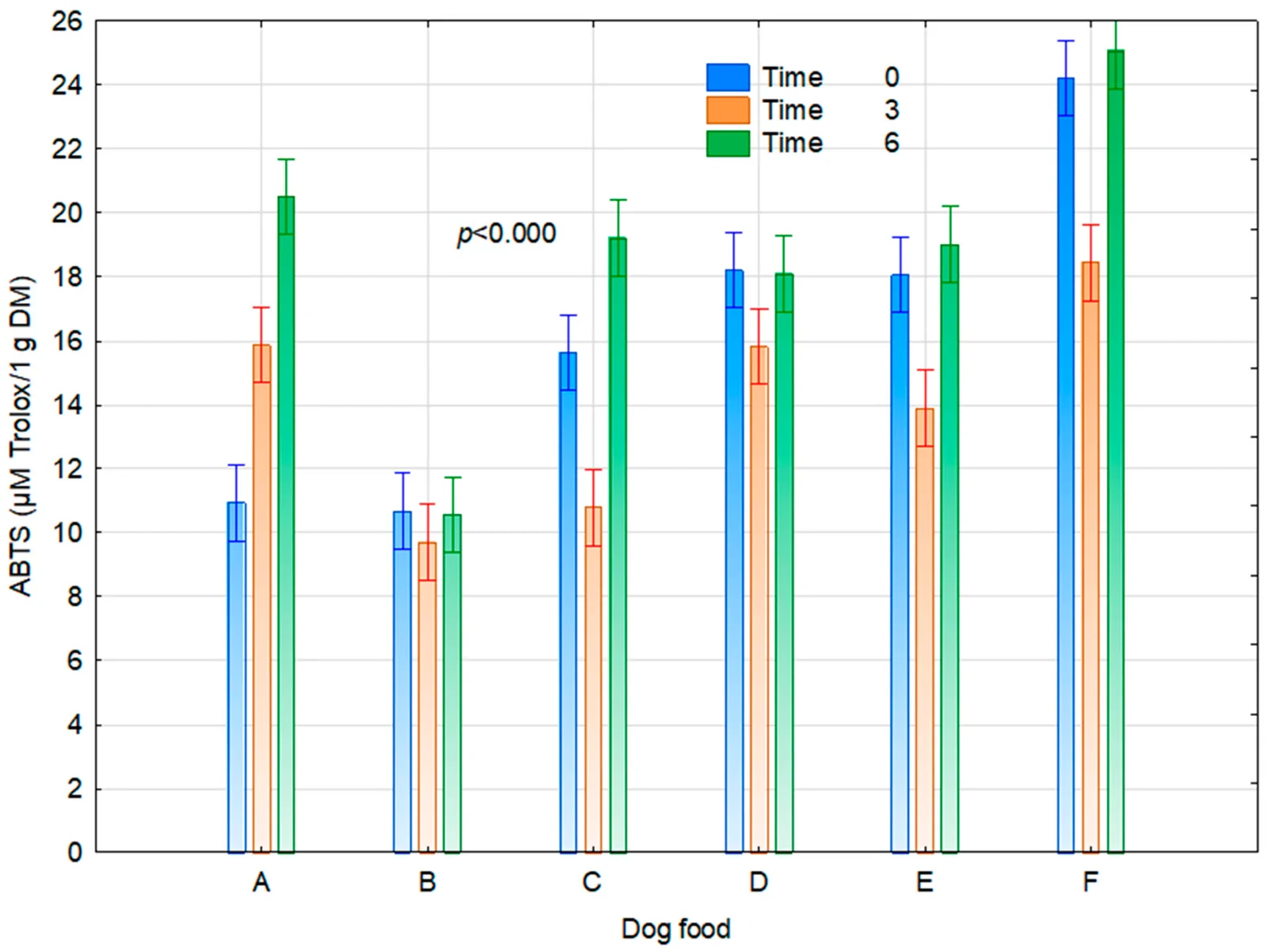
The ABTS (µM Trolox/1 g DM) variations in dog foods over time (0, 3, and 6 months after opening); the main animal protein sources declared by the manufacturers were lamb (food A), poultry (food B), insects (food C), fish (foods D and F), and pork (food E); replicate number = 3.

**Figure 22:**
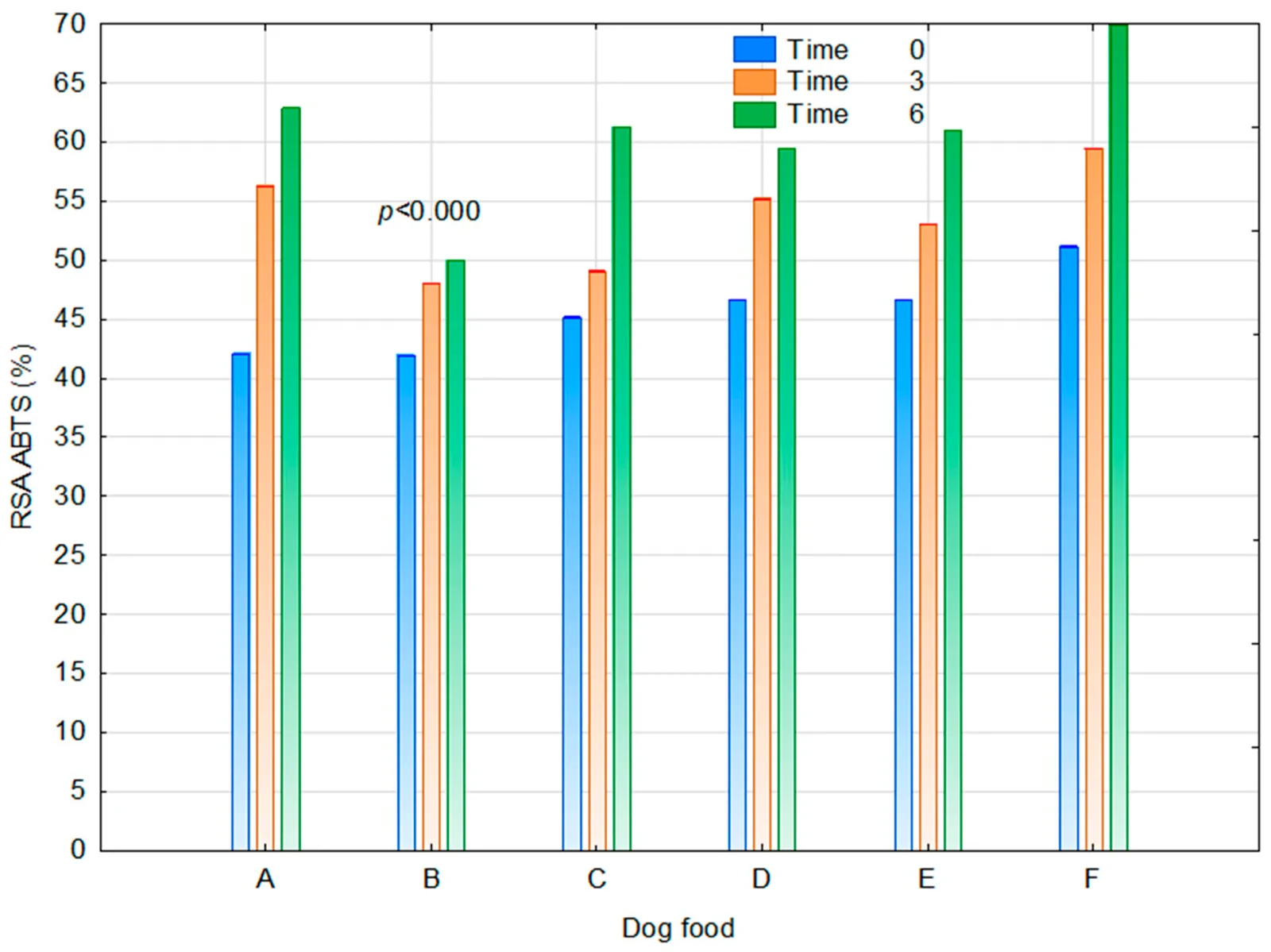
The RSA ABTS (%) variations in dog foods over time (0, 3, and 6 months after opening); the main animal protein sources declared by the manufacturers were lamb (food A), poultry (food B), insects (food C), fish (foods D and F), and pork (food E); replicate number = 3.

Detailed values related to the antioxidant properties of individual foods at all stages of analysis were statistically significant and are presented in Figure 19, Figure 20, Figure 21 and Figure 22.

**Figure 23:**
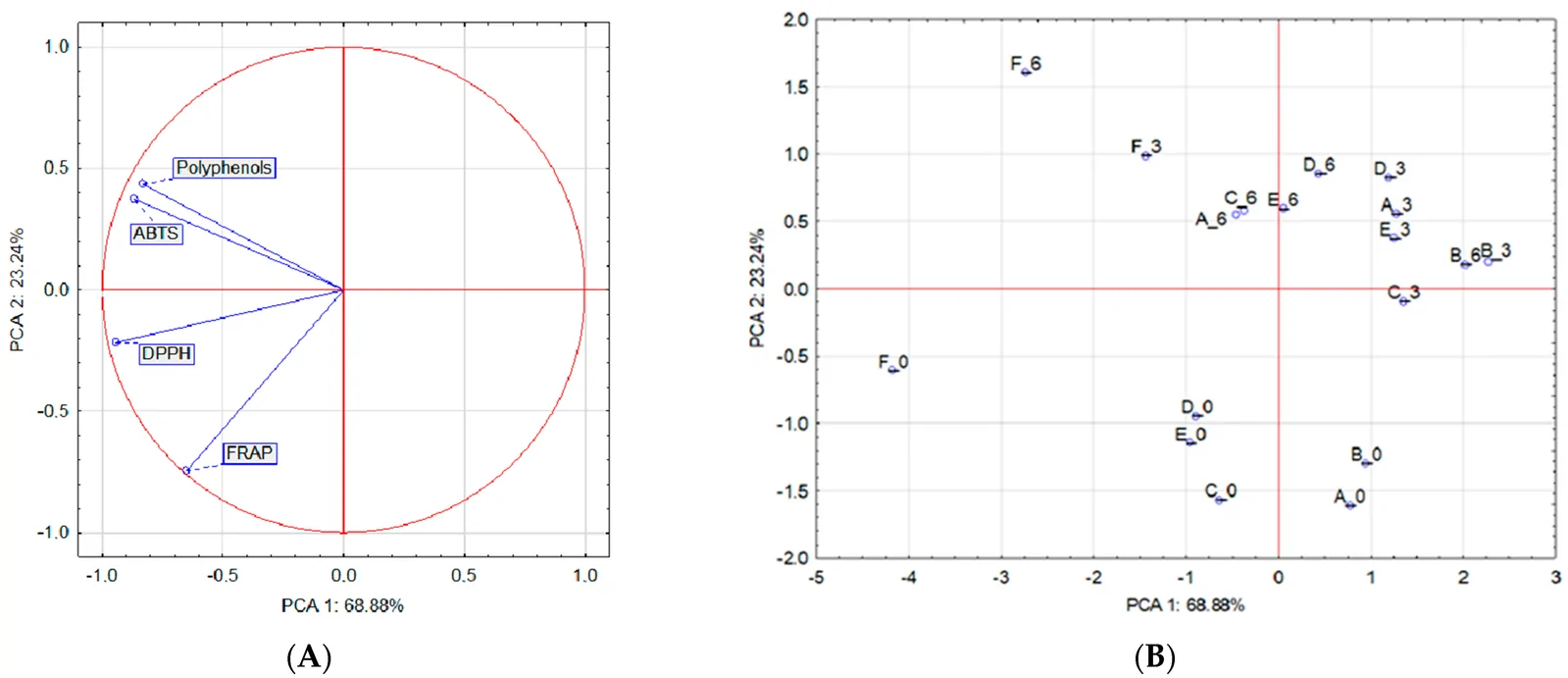
Biplot based on the first two principal component axes for polyphenol content and antioxidant properties of dog foods (**A**) and the distribution of six dog foods based on these components (**B**); the main animal protein sources declared by the manufacturers were lamb (food A), poultry (food B), insects (food C), fish (foods D and F), and pork (food E); replicate number = 3.

In the case of polyphenol content and antioxidant properties, PCA analysis showed that the first two components account for 92.12% of the total variance (Figure 23A). This analysis clearly shows that the polyphenol content and antioxidant properties of dog foods at the time of opening the packages are very similar; these products are located in the second quadrant of the coordinate system. The second separate group consists of foods whose packaging was opened 3 and 6 months ago (Figure 23B).

## 3. Discussion

### 3.1. Chemical Composition and Nutrient Stability

All analyzed foods met the MRL in case of protein and fat [2]. Over the six months of storage, changes were observed in the content of dry matter and crude fat in the foods. The decrease in dry matter is due to the inevitable access of moisture to the food after opening the package, which results in an increase in the moisture content of the food itself.

### 3.2. Antioxidant Stability and Its Role in Dog Food Preservation

Dry dog foods are generally more stable than wet foods due to their lower water content, which allows for longer storage. However, dry foods may still require the addition of antioxidants to prevent rancidity, a process primarily affecting polyunsaturated fatty acids (PUFA). The oxidation of PUFA leads to the formation of harmful by-products, including aldehydes and ketones, which can impair nutritional value, taste, and aroma, and it decreases shelf life [10,15]. This is particularly relevant in the production of commercially available complete dry pet foods, where selected ingredients undergo high-temperature extrusion prior to final formulation and drying [16].

To counteract oxidation, antioxidants—either synthetic or natural—are added. However, recent studies suggest a growing concern about the potential harmful effects of synthetic antioxidants, such as butylated hydroxytoluene (BHT) and butylated hydroxyanisole (BHA), which have been linked to hepatotoxicity and carcinogenicity [17,18,19]. In light of these risks, there is increasing interest in using natural antioxidants derived from plant sources, including vitamins A, C, E, carotenoids, and polyphenols [20,21]. These compounds have been shown to improve oxidative status and may even offer health benefits to dogs by scavenging free radicals and supporting immune function [22,23]. For instance, vitamin C, found in citrus fruits and parsley, and vitamin E, present in vegetable oils, can protect cellular structures from oxidative damage [23,24].

Studies on the impact of antioxidants on dogs’ health show that short-term supplementation can delay age-related declines and alleviate symptoms of oxidative stress, such as in neurodegenerative diseases [25]. Furthermore, antioxidants help balance the effects of reactive oxygen species (ROS), which are generated during normal metabolism and contribute to a variety of age-related diseases in both humans and animals [21]. Additionally, antioxidant capacity in foods can be evaluated using methods like ferric reducing antioxidant power (FRAP) and oxygen radical absorbance capacity (ORAC), which measure the ability of foods to neutralize free radicals [26,27].

Lipid oxidation is a key factor in the deterioration of food quality, leading to off-flavors, loss of nutrients, and reduced shelf stability [28]. Some plant extracts, such as thyme and rosemary, have been shown to effectively inhibit lipid oxidation in food products due to their high concentrations of phenolic compounds [29]. Studies have demonstrated that dogs fed diets supplemented with natural antioxidants, such as a blend of rosemary, oregano, and vitamin E, exhibit reduced levels of ROS, highlighting the importance of antioxidant-rich diets for maintaining both food quality and animal health [30].

Notably, during the storage period, fluctuations were observed in both the total polyphenol content and the antioxidant properties (DPPH, FRAP, ABTS) of the analyzed foods. These changes may be attributed to the complex interactions between polyphenolic compounds and the food matrix, as well as the potential degradation or transformation of phenolic structures under ambient storage conditions. Decreases in polyphenol content and antioxidant properties after three months may reflect oxidative degradation of labile phenolic compounds or volatilization of active antioxidant components, particularly in foods rich in herbal and fruit extracts. This trend was observed in foods B, C, D, and E. In food B, the potential source of phenolic compounds was rosemary extract only. In the case of food C, it was seaweed. The situation of foods D and E is slightly more interesting, as they were rich in potential sources of phenolic compounds. In the case of food D, they were fresh cranberries, fresh blueberries, chicory root, turmeric, milk thistle, burdock root, lavender flower, marshmallow root, rose hips. In food E, they were sea algae, fresh whole cranberries, fresh whole blueberries, chicory root, turmeric, milk thistle, burdock root, lavender flower, marshmallow root, and rosehips. The manufacturer did not provide the amounts used, but the power of these antioxidants could have been too weak to maintain a constant polyphenol content during product storage.

Interestingly, an increase in polyphenol content and antioxidant properties after six months was also observed in some cases. This phenomenon may be related to the release of bound polyphenols from fiber–protein complexes or the formation of new antioxidant compounds as a result of Maillard reaction products or lipid oxidation by-products. Such non-enzymatic browning reactions can produce compounds with radical scavenging capacity [31,32,33].

### 3.3. Changes in the Fatty Acid Composition During Storage

The content and proportions of fatty acids in dog foods, especially essential polyunsaturated fatty acids (PUFA), are critical for maintaining nutritional quality. The FEDIAF Nutritional Guidelines [2] specify MRL for linoleic acid (C18:2 n-6) but do not provide guidance for other fatty acids. In this study, all foods met the MRL for linoleic acid despite some observed losses over six months of storage. For example, food A, which used canola oil as a fat source, showed notable changes in the fatty acid profile during storage, with a decrease in the saturated fatty acid (SFA) and palmitic acid (C16:0) and an increase in oleic acid (C18:1 n-9) and linoleic acid (C18:2 n-6) levels. Similar results were obtained by Hołda and Głogowski [14], who observed a decrease in SFA and a significant increase in C18:1 n-9 saturated fatty acid content after 7 months of storage of dog foods under typical conditions. These authors suggested that one of the probable explanations may be the reactivation of plant lipases, related to the inevitable increase in moisture after opening the packages and during storage at room temperature.

Similarly, food B, in which the manufacturer declares poultry fat as the main source of fat, showed significant changes in fatty acid concentrations, particularly in oleic acid (C18:1 n-9) and linoleic acid (C18:2 n-6). The addition of rosemary extract, a natural antioxidant, did not completely prevent these changes, indicating that more potent or higher concentrations of antioxidants may be needed to stabilize fatty acids in foods with high fat content. In turn, in foods D and F, which contained fish oils, the expected high levels of EPA and DHA were not consistently maintained during storage. While food F had the highest antioxidant properties, changes in the fatty acid composition during storage remained a concern. The loss of essential n-3 fatty acids in these foods is particularly significant given their importance for brain and eye health [34,35]. This loss could be attributed to the high susceptibility of fish oils to oxidation, which may be exacerbated by inadequate antioxidant levels. In the research of Hołda and Głogowski [14] of the long-chain polyunsaturated fatty acids typically found in fish, only DHA was detected in the lipid fraction. In one food, a particularly high DHA content was detected when freshly opened, and it decreased significantly during storage. Ahlstrøm et al. [36] observed significant variations in the fatty acid content of commercial dry dog foods (mainly puppy foods), suggesting that the absence of DHA or EPA practically reflects the absence of marine oils or animal products in the kibble.

The observed increases in the relative concentrations of n-3 and n-6 fatty acids after storage likely reflect the selective degradation of other lipid fractions or matrix-related redistributions rather than true synthesis. Hołda and Głogowski [14] observed an increase in particular unsaturated fatty acids, during storage, in dry dog foods. The authors suggested that reactivated lipase activity, possibly due to increased humidity, may have altered the lipid profile, reducing certain components and thus raising the relative percentage of more stable fatty acids like oleic acid. Another study examined changes in fatty acid profiles of edible oils commonly used in homemade pet diets over 12 months of storage. It revealed that peroxide value increased significantly in most samples, while induction time (a measure of oxidative stability) declined. Although absolute losses in linoleic acid were noted, the storage effects underscore that degradation and oxidative changes can alter both absolute and relative lipid composition [37].

Additionally, research on commercial dog foods (both wet and dry) stored at varying temperatures (up to + 40 °C) over 12 months showed that oxidative markers rose with time and temperature, and EPA + DHA contents decreased up to 50% in economic-class dry foods. This confirms that PUFAs are particularly prone to oxidation, influencing the overall fatty acid balance [38].

The oxidative degradation of PUFA is markedly influenced by storage conditions, particularly temperature, exposure to light, and oxygen availability. Marine-derived oils rich in EPA and DHA are especially vulnerable to lipid peroxidation. Studies show that even refrigeration may not sufficiently halt oxidation beyond a month, while exposure to heat, light, and oxygen accelerates the formation of primary (hydroperoxides) and secondary oxidation products (e.g., aldehydes like HHE and MDA) [39]. For example, research on hoki liver oil demonstrated that exposure to ambient air and light rapidly elevated peroxide values compared to thermal stress in the dark; yet, after prolonged storage, both conditions resulted in similar oxidation levels, underscoring the significance of even subtle environmental factors [40]. Modeling of oxidation kinetics in commercial fish oils confirmed that higher PUFA content correlates with increased susceptibility; the elevation of storage temperature above 40 °C disrupts first-order kinetic assumptions, highlighting the need for controlled conditions during accelerated testing [41]. Long-term storage at elevated temperatures leads to significant modifications in fatty acid composition. For instance, peanuts stored at 15 °C, 25 °C, and 35 °C over 320 days showed rising peroxide, carbonyl, and MDA levels; furthermore, unsaturated fatty acids, especially linoleic and linolenic, decreased, while oleic acid proportionally increased [42].

Studies show that both the storage conditions and the formulation of pet food play a crucial role in maintaining its safety and nutritional value. Survey data from pet caregivers indicate that dry food is typically purchased in bulk packages, lasting a minimum of four weeks, and it is frequently stored in plastic containers in the kitchen, sometimes under conditions that may promote higher temperatures. Furthermore, 67.3% of those feeding commercial diets regard preservatives as a possible health concern, while a substantial proportion of homemade diet feeders keep temperature-sensitive ingredients, such as fish oil, at ambient conditions, potentially accelerating their deterioration [11]. Research by Usuga et al. [12] demonstrated that dry diets can influence the oxidative–antioxidant status of dogs’ blood, although this effect does not appear to correlate directly with the measured antioxidant levels in the diets themselves. These findings emphasize the importance of informing pet caregivers about appropriate storage practices and clarifying the functional role of technological additives—such as antioxidants and preservatives—in ensuring both shelf life and product safety.

The n-6/n-3 fatty acid ratio is a crucial factor influencing inflammation and overall health in dogs. A ratio of 5:1 to 10:1 has been associated with reduced inflammation, while higher ratios (e.g., 100:1) can promote inflammatory responses [43]. In our study, the n-6/n-3 ratio ranged from 0.93:1 in food D to 58.91:1 in food C, indicating significant variations in fatty acid composition among the different foods. The average n-6/n-3 ratio increased from 18.11:1 at the time of opening to 29.53:1 after 3 months and 24.01:1 after 6 months. These changes suggest that the optimal ratio of these fatty acids was not consistently maintained, which could have implications for long-term health, especially in terms of reducing the risk of inflammatory diseases like osteoarthritis and cancer [44]. Diets with lower ratios, such as D and F (0.93 and 3.15, respectively), are more favorable for controlling systemic inflammation and may support healthier lipid profiles in dogs, as shown by Sechi et al. [45], who reported that higher n-3 levels were associated with increased circulating DHA and EPA and improved lipid metabolism in aged dogs. The h/H ratio indicates the potential of a diet to modulate cholesterol metabolism. Higher values are preferred, as they suggest a greater proportion of fatty acids that can reduce total and LDL cholesterol. In our research, food A (5.55) showed the most favorable value, likely due to its high MUFA content from canola oil, while food C (0.78) presented less advantageous profiles. In canine nutrition, higher h/H ratios might be associated with reduced serum total cholesterol and LDL concentrations, which could lower the risk of obesity-related dyslipidemia. Obese dogs often exhibit altered lipid and metabolic profiles, including elevated triglycerides and VLDL, which are associated with more aggressive tumors. This suggests a link between obesity-induced dyslipidemia and health complications in dogs [46]. DFA is linked to beneficial effects on lipid metabolism and cell membrane function. Values above 70% are generally considered favorable. Most foods tested, except for C (48.34), showed high DFA contents, with A (84.73) being the most favorable. Diets rich in DFA, i.e., monounsaturated and polyunsaturated fats, have been shown to alter canine plasma lipids and lipoprotein distributions, and in clinical cases, n-3 supplementation reduces triglyceride and total cholesterol effects that are consistent with maintaining HDL and limiting excessive triglyceride accumulation in tissues [47]. Direct evidence for reduced adipose triglyceride accumulation in dogs is limited; however, studies in other species show that dietary fatty acid composition influences adipose ‘trapping’ of dietary fats. Conversely, OFA, representing atherogenic saturated fatty acids (mainly lauric, myristic, and palmitic acids), should be as low as possible. Foods C (50.39%) and B (26.23%) displayed the highest OFA values, suggesting a less favorable lipid profile for long-term consumption, while A (13.85) and F (17.28) were more aligned with cardiovascular and metabolic health goals. Elevated OFA intake in dogs could contribute to hyperlipidemia and higher circulating VLDL concentrations, but research is needed in this direction. Furthermore, fatty acid composition plays a role in modulating various health indices, including the index of atherogenicity (IA), which assesses the potential for a food to contribute to atherosclerosis. A lower IA value signifies a more favorable balance between SFAs and unsaturated fatty acids, which could support cardiovascular health and reduce the risk of inflammatory diseases [48]. The atherogenic index and thrombogenic index provide further insight into the potential impact of dietary fatty acids on cardiovascular health. Lower values are preferred. AI values ranged from 0.22 (food A) to 1.63 (food C), and TI values ranged from 2.28 (food C) to 29.34 (food D). Diet C presented both the highest AI and one of the lowest h/H values, indicating a less protective profile against lipid-related disorders. In contrast, food A, with the lowest AI and TI, represents the most favorable combination for reducing atherogenic and thrombogenic risks in dogs. While the link between these indices and clinical cardiovascular events in dogs is less direct than in humans, elevated AI and TI have been correlated with increased plasma triglycerides and LDL, particularly in obese or aging dogs [45].

## 4. Material and Methods

### 4.1. Material

The research material of this pilot study consisted of six commercial mono-protein dry complete foods for adult dogs. The ingredient compositions of these foods are presented in Table S1. The main animal protein sources declared by the manufacturers were lamb (food A), poultry (food B), insects (food C), fish (foods D and F), and pork (food E). The fat sources were canola oil (food A), poultry fat and sunflower oil (food B), insect oil (food C), pollack oil and cold-pressed sunflower oil (food D), pork fat (food E), and olive oil (food F). These foods were selected based on the presence of specific plant-based ingredients (e.g., berries, herbs, or vegetables) listed on the label, which are commonly associated with high phenolic content.

Food A contained dried chicory root, blueberries, and raspberries. Food B included rosemary extract. Food C contained seaweed. Food D was enriched with fresh cranberries, fresh blueberries, chicory root, turmeric, milk thistle, burdock root, lavender flower, marshmallow root, and rose hips. Food E featured sea algae, fresh cranberries, fresh blueberries, chicory root, turmeric, milk thistle, burdock root, lavender flower, marshmallow root, and rose hips. Food F was the most diverse, containing dried cranberry, chamomile powder, burdock root powder, anise, fenugreek, peppermint, calendula, grape seed extract, green tea extract, dried dandelion, dried blueberries, ginseng, thyme, marjoram, oregano, parsley, sage, and other herbs.

The foods were purchased from Polish pet stores in December 2022. Although purchased locally, most brands were international. The bags, ranging in weight from 0.5 kg to 3 kg, were produced in the fourth quarter of 2022 and opened on 10 December 2022. Each food trial consisted of three packages of the same product. A representative 100 g sample of each food was taken from each package for laboratory analysis. Food samples were collected for analysis from the bottom, middle, and top of the package immediately after opening and after three and six months of storage at ambient laboratory conditions (temperature: 20 °C, humidity: 40%). They were combined and thoroughly mixed to obtain the laboratory sample. Then, the material for analysis was collected from the laboratory sample, which constituted the analytical sample [49,50].

Samples from each food were ground using a KNIFETEC 1095 laboratory mill (0.75 mm; Foss Tecator, Höganäs, Sweden), placed in sterile containers, and labeled as A–F [49]. The analyses were performed in triplicate at three time points: upon opening (December 2022), after 3 months (March 2023), and after 6 months (June 2023).

### 4.2. Chemical Composition

The proximate composition was determined in the ground samples according to the Association of Official Analytical Chemist methods [51]. To determine dry matter (DM), samples were dried at 105 °C to a constant weight (and content of moisture—method 945.15). Crude protein (CP) (method 945.18) was determined from 6.25 × total nitrogen measured by the Kjeldahl method using a Büchi Scrubber B414 digestion apparatus and a Büchi 324 distillation set (Büchi Labortechnik AG, Flawil, Switzerland). Crude fat (as ether extract, EE) was determined by the Soxhlet method, with diethyl ether used as a solvent (method 2003.06). The crude fiber (CF) was determined with an ANKOM^220^ Fiber Analyzer (ANKOM Technology, New York, NY, USA) (method 962.09). Crude ash (CA) was determined by burning in a muffle furnace in 580 °C for 8 h (method 920.153). All analyses were performed in duplicate. Nitrogen-free extracts (NFE) were calculated using the formula: NFE: 100—(moisture content + CP + EE + CA + CF). All results were expressed in grams per 100 g of dry matter (g/100 g DM).

### 4.3. Calculation of Metabolizable Energy

The metabolizable energy (ME) value of dog foods was calculated on the basis of the determined proximate composition. According to FEDIAF [2], for the calculation of ME in prepared pet foods for dogs, the following four steps have been used:

Calculate gross energy (GE):

GE (kcal) = (5.7 × %CP) + (9.4 × %EE) + [4.1 × (%NFE + %CF)](1)

Calculate energy digestibility (ED) (%):

ED (%) = 91.2 − (1.43 × %CF in DM)(2)

Calculate digestible energy (DE):

DE (kcal) = (kcal GE × ED)/100(3)

Calculate metabolizable energy (ME):

ME (kcal) = DE − (1.04 × %CP)(4)

### 4.4. Fatty Acid Content

The fatty acid composition of the samples was determined using a modified Folch method. Approximately 3 g of each sample was homogenized with 25 mL of a chloroform (Sigma-Aldrich, St. Louis, MO, USA)–methanol (Sigma-Aldrich, St. Louis, MO, USA) mixture in a ratio of 2:1 (*v*/*v*). The mixture was filtered, and 5 milliliters of the filtrate was combined with 1 mL of 0.58% sodium chloride solution (Chempur, Piekary Śląskie, Poland) to separate the phases. After centrifugation at 2500 revolutions per minute for 10 min, the chloroform layer containing the lipids was collected and evaporated to dryness under a stream of nitrogen at 65 °C, leaving a lipid residue. The lipids were subjected to hydrolysis by adding 3 mL of 0.5 molar sodium hydroxide (Chempur, Piekary Śląskie, Poland) in methanol (Sigma-Aldrich, St. Louis, MO, USA), followed by incubation in a water bath at 75 °C for 20 min. After cooling, the hydrolyzed samples were esterified by adding 3 mL of boron trifluoride (BF_3_) (Sigma-Aldrich, St. Louis, MO, USA) in methanol and heating at 75 °C for 10 min. The resulting fatty acid methyl esters were extracted with 2 mL of heptane, dried over sodium sulphate, and stored for analysis. The fatty acid profile was analyzed using gas chromatography with a flame ionization detector (GC-FID) (Varian, Palo Alto, CA, USA). The analyses were performed on a Varian 450-GC (Varian, Palo Alto, CA, USA) equipped with an Rt-2560 capillary column (100 m × 0.25 mm × 0.20 μm) (Restek Corporation, Bellefonte, PA, USA). Helium (Linde Gaz Polska Sp. z o.o., Cracow, Poland; high-purity grade) was used as the carrier gas at a flow rate of 1 mL/min. The injection volume was 1 microliter, with the injector set at 200 °C and the detector at 250 °C. The column temperature was programmed to increase from 60 °C to 220 °C.

### 4.5. Index of Atherogenicity (IA)

The atherogenic potential of FAs was determined using the index of atherogenicity (IA) [52], according to the following Equation (5):

(5) $$IA=\frac{C12\text{:}0+\left(4\times C14\text{:}0\right)+C16\text{:}0}{\Sigma UFA}$$

### 4.6. Index of Thrombogenicity (IT)

The thrombogenic potential of FAs was determined using the index of thrombogenicity (IT) [52], according to the following Equation (6):

(6) $$IT=\frac{C14\text{:}0+C16\text{:}0+C18\text{:}0}{\left(0.5\times \Sigma MUFA\right)+\left(0.5\times \Sigma n-6\;PUFA\right)+\left(3\times \Sigma n-3\;PUFA\right)+(n-3/n-6)}$$

### 4.7. Index of Hypocholesterolemic/Hypercholesterolemic Acids (h/H)

The h/H index of fatty acids is a measure used in lipid chemistry to quantify the degree of unsaturation (double bonds) and to give insight into the distribution of fatty acid chain lengths and their specific positions. The h/H index was determined according to the following Equation (7):

(7) $$h/H=\frac{\left(C18\text{:}1\;n-9\;cis+C18\text{:}2\;n-6\;cis+C18\text{:}3\;n-3\right)}{\left(C12\text{:}0+C14\text{:}0+C16\text{:}0\right)}$$

### 4.8. Hypocholesterolemic Acids (DFA) and Hypercholesterolemic Acids (OFA)

The terms DFA and OFA refer to different classes of fatty acids based on their effects on cholesterol levels in the body. They were determined according to the following Equations (8) and (9):

(8) DFA=UFA+C18:0

(9) OFA=C12:0+C14:0+C16:0

### 4.9. Determination of Total Phenolic Compound Content

In order to measure the total content of phenolic compounds, methanol extracts were used using the Folin–Ciocalteau reagent. This method involves the colorimetric determination of colored products, which are formed when polyphenolic compounds react with the Folin–Ciocalteau reagent (Sigma, St. Luis, Missouri, MO, USA). The concentration of total polyphenolic compounds was spectrophotometrically determined at a wavelength of λ = 760 nm using a RayLeigh UV-1800 spectrophotometer (Beijing Beifen-Ruili Analytical Instrument, Beijing, China), according to the Folin–Ciocalteau method [53]. The results were expressed as chlorogenic acid equivalents (CGA) (Merck, Darmstadt, Germany) in milligrams per 100 g of dry matter based on a standard curve. The total polyphenol content was expressed as gallic acid equivalents (GAE), based on a calibration curve prepared using gallic acid as a standard.

### 4.10. Determination of Antioxidant Properties

#### 4.10.1. ABTS Method

Antioxidant properties were evaluated using methanol extracts to determine the sample’s ability to scavenge free radicals, specifically ABTS (2,2′-azinobis-(3-ethylbenzothiazoline-6-sulfonic acid)) (Merck, Darmstadt, Germany), following the method of Prior et al. [54]. Absorbance was measured at 734 nm using a Rayleigh UV-1800 spectrophotometer (Beijing Beifen-Ruili Analytical Instrument, Beijing, China). The results for each sample were compared with the standard Trolox concentration–response curve and expressed as micromoles of Trolox equivalents per gram of DM.

#### 4.10.2. DPPH Method

The DPPH test was conducted based on the method of Miliauskas et al. [55] with modifications. A stock solution of DPPH was prepared by dissolving 6 mg of 2,2-diphenyl-1-picrylhydrazyl (Merck, Darmstadt, Germany) in 100 mL of methanol. The working solution was prepared by diluting the stock solution with methanol to achieve an absorbance of 0.900–1.000 at 515 nm. A volume of 80 µL of the extract was transferred into a test tube and diluted to 1.5 mL with methanol. After mixing the diluted extract with 3 mL of the DPPH solution, the mixture was incubated in the dark at room temperature for 10 min. Absorbance at 515 nm was then recorded. The results for each sample were compared to the Trolox standard concentration–response curve and expressed as micromoles of Trolox equivalents per gram of DM.

#### 4.10.3. FRAP Method

The total reducing capacity of the samples was determined using the FRAP method, as described by Benzie and Strain [26], with modifications. The FRAP reagent was prepared by mixing 100 mL of 300 mM acetate buffer (pH 3.6), 10 mL of 10 mM TPTZ (2,4,6-tris(2-pyridyl)-s-triazine) (Merck, Darmstadt, Germany) in 40 mM hydrochloric acid, and 10 mL of 20 mM ferric chloride hexahydrate. A volume of 10–200 µL of the extract was transferred into a test tube and diluted to 1 mL with 70% methanol. After mixing the diluted extract with 3 mL of the FRAP (Merck, Darmstadt, Germany) reagent, the mixture was incubated in the dark at room temperature for 10 min. Absorbance was measured at 593 nm using the Rayleigh UV-1800 spectrophotometer (Beijing Beifen-Ruili Analytical Instrument, Beijing, China). Results were compared to the Trolox standard concentration–response curve and expressed as micromoles of Trolox equivalents per gram of dry matter.

The percentage inhibition of DPPH and ABTS radical generation was calculated using the following Formula (10):

RSA% = [(A0 − A1)/A0] × 100(10)

where RSA is the radical scavenging activity, A0 is the absorbance of the control (for ABTS) and of the sample at the beginning of the reaction (for DPPH), and A1 is the absorbance of the sample after 6 min (for ABTS) or 10 min (for DPPH) [56].

### 4.11. Statistical Analyses

The obtained results were statistically analyzed using the Statistica 13.3 software [57]. A two-way analysis of variance (ANOVA) was performed for all analyzed variables to assess the effects of time (after opening, 3 months, and 6 months of storage) and in dog food. Principal component analysis (PCA) was also conducted to explore patterns and relationships in the data. Tukey’s honestly significant difference (HSD) at *p* ≤ 0.05 was used to find the differences between means.

## 5. Conclusions

The research demonstrates that the quality of commercial dry dog foods, particularly regarding oxidative stability and antioxidant properties, is subject to change over time after opening (*p ≤* 0.05). The chemical composition of the foods was influenced by storage duration, with significant decreases in crude fat and variations in fatty acid profiles (*p* ≤ 0.05). The study highlighted the importance of antioxidant additives in maintaining food quality, as evidenced by the fluctuating polyphenol content and antioxidant properties. PCA analysis revealed that each food’s fatty acid and antioxidant profile varied over time, indicating the need for the careful monitoring of food composition to ensure it remains nutritionally adequate and safe for pets. The findings also highlighted that the antioxidants currently used in dry dog foods may be insufficient in many cases to maintain the oxidative stability of the products during up to six months of storage. Therefore, this research underscores the necessity of enhancing antioxidant strategies, including compound selection, concentration, and timing of application or exploring alternative preservation methods to ensure the long-term stability of dry dog foods. Diets with adequate antioxidant supplementation—preferably using natural antioxidants like vitamin E, polyphenols, or plant extracts—may offer better protection against lipid oxidation and support overall health. This pilot study was limited by the relatively small number of tested products, which may not represent the full diversity of commercial dry dog foods. The analysis focused solely on foods available in the European market, which may limit the applicability of the findings to other regions. Additionally, storage was evaluated under typical household conditions, and different storage conditions could influence oxidative stability and antioxidant properties.

## Figures and Tables

**Table 1:** Proximate composition (g/100 g DM) and metabolizable energy (kcal/100 g DM) of the analyzed dog foods after opening the packages.

Dog Food	Dry Matter (g/100 g of Fresh Matter)	Crude Protein	Nitrogen-Free Extracts	Crude Ash	Crude Fiber	Metabolizable Energy
A	94.17 ^abc^	29.68 ^b^	38.45 ^c^	6.91 ^a^	5.62 ^c^	398.2 ^a^
B	94.56 ^bc^	41.41 ^e^	27.19 ^a^	8.36 ^a^	4.19 ^ab^	382.6 ^a^
C	93.76 ^abc^	26.67 ^a^	47.04 ^d^	6.02 ^a^	6.09 ^c^	384.9 ^a^
D	91.42 ^a^	39.87 ^e^	30.04 ^ab^	7.80 ^a^	4.17 ^ab^	383.4 ^a^
E	92.19 ^ab^	34.44 ^c^	37.05 ^bc^	6.96 ^a^	5.21 ^bc^	384.8 ^a^
F	95.44 ^c^	36.47 ^d^	26.94 ^a^	9.45 ^a^	3.78 ^a^	388.4 ^a^

DM—dry matter; means with at least one of the same letters in the superscript (a, b, c, d, e) do not differ statistically at *p* ≤ 0.05 (for all columns separately); the main animal protein sources declared by the manufacturers were lamb (food A), poultry (food B), insects (food C), fish (foods D and F), and pork (food E).

**Table 2:** Average dry matter (g/100 g of fresh matter) and crude fat (g/100 g of DM) content in dog foods after opening the packages.

Dog Food	Dry Matter (g/100 g of Fresh Matter)	Crude Fat (g/100 g of DM)
A	94.17 ^abc^	19.35 ^e^
B	94.56 ^bc^	18.86 ^d^
C	93.76 ^abc^	14.17 ^a^
D	91.42 ^a^	18.12 ^c^
E	92.19 ^ab^	16.34 ^b^
F	95.44 ^c^	23.37 ^f^
**Time After Opening (Months)**		
0	93.59 ^b^	18.37 ^c^
3	92.89 ^a^	17.88 ^b^
6	95.04 ^c^	16.87 ^a^

DM: dry matter; means with at least one of the same letters in the superscript (a, b, c, d, e, f) do not differ statistically at *p* ≤ 0.05 (for all columns separately); the main animal protein sources declared by the manufacturers were lamb (food A), poultry (food B), insects (food C), fish (foods D and F), and pork (food E).

**Table 3:** Average saturated fatty acid (SFA) content (% of total fatty acids) over time and averages for all dog foods after opening the packages.

Dog Food	C4:0	C6:0	C8:0	C10:0	C11:0	C12:0	C13:0	C14:0	C15:0	C16:0	C17:0	C18:0	C20:0	C21:0	C22:0	C23:0	C24:0	SFA
A	0.00	0.01	0.03	0.02 ^ab^	0.01	0.19 ^bc^	0.01	1.01 ^a^	0.15 ^a^	12.66 ^a^	0.31 ^bc^	5.97 ^d^	0.38 ^e^	0.00	0.12	0.22 ^b^	0.07 ^a^	21.14 ^a^
B	0.00	0.02	0.01	0.03 ^ab^	0.00	0.44 ^d^	0.05	1.82 ^c^	0.23 ^b^	23.96 ^e^	0.40 ^e^	7.89 ^e^	0.11 ^a^	0.00	0.14	0.32 ^c^	0.02 ^a^	35.45 ^d^
C	0.00	0.00	0.00	0.49 ^c^	0.01	25.47 ^e^	0.04	6.93 ^f^	0.21 ^b^	17.99 ^d^	0.17 ^a^	3.97 ^b^	0.12 ^a^	0.01	0.04	0.05 ^a^	0.03 ^a^	55.54 ^f^
D	0.00	0.00	0.05	0.01 ^a^	0.01	0.23 ^c^	0.02	4.08 ^e^	0.29 ^c^	14.62 ^b^	0.27 ^b^	3.69 ^a^	0.28 ^c^	0.00	0.00	0.27 ^bc^	3.56 ^d^	27.37 ^c^
E	0.01	0.01	0.01	0.04 ^ab^	0.01	0.15 ^ab^	0.01	1.58 ^b^	0.12 ^a^	24.12 ^f^	0.37 ^de^	13.30 ^f^	0.18 ^b^	0.00	0.00	0.29 ^bc^	0.25 ^b^	40.45 ^e^
F	0.00	0.01	0.02	0.06 ^b^	0.01	0.10 ^a^	0.01	2.42 ^d^	0.23 ^b^	14.76 ^c^	0.33 ^cd^	4.73 ^c^	0.31 ^d^	0.01	0.00	0.24 ^b^	1.75 ^c^	25.04 ^b^
**Time After Opening (Months)**																		
0	0.01	0.01	0.03	0.08 ^a^	0.01	2.91 ^a^	0.05	3.26 ^c^	0.32 ^b^	20.72 ^c^	0.42 ^b^	7.61 ^b^	0.19 ^a^	0.01	0.05	0.26 ^b^	0.62 ^a^	36.57 ^c^
3	0.00	0.01	0.02	0.12 ^b^	0.01	4.98 ^b^	0.01	2.79 ^a^	0.14 ^a^	16.75 ^b^	0.24 ^a^	6.13 ^a^	0.25 ^b^	0.02	0.04	0.25 ^b^	1.09 ^b^	32.83 ^a^
6	0.00	0.01	0.02	0.13 ^b^	0.01	5.40 ^c^	0.02	2.87 ^b^	0.16 ^a^	16.57 ^a^	0.27 ^a^	6.03 ^a^	0.26 ^b^	0.01	0.05	0.19 ^a^	1.13 ^c^	33.10 ^b^

SFA—saturated fatty acids; means with at least one of the same letters in the superscript (a, b, c, d, e, f) do not differ statistically at *p* ≤ 0.05 (for all columns separately); the main animal protein sources declared by the manufacturers were lamb (food A), poultry (food B), insects (food C), fish (foods D and F), and pork (food E).

**Table 4:** Average unsaturated fatty acid (UFA) (% of the sum of fatty acids) content in dog foods after opening the packages.

Dog Food	C14:1 (n-9)	C15:1	C16:1 (n-9)	C17:1 (n-9)	C18:1 (n-9 cis)	C20:1 (n-9)	C22:1 (n-9)	C24:1	C18:2 (n-6 trans)	C18:2 (n-6 cis)	C20:2 (n-9)	C22:2 (n-6)	C18:3 (n-3)	C18:3 (n-6)	C20:3 (n-3)	C20:3 (n-6)	C20:4 (n-6)	C20:5 (n-3)	C22:6 (n-3)	Unidentified
A	0.05 ^b^	0.04	1.39 ^a^	0.18 ^ab^	53.79 ^f^	4.67 ^f^	0.05	0.04	0.07	16.92 ^d^	0.06	0.04 ^ab^	0.94 ^d^	0.16	0.02	0.22 ^c^	0.01 ^a^	0.03 ^b^	0.07 ^ab^	0.11 ^a^
B	0.17 ^d^	0.00	3.97 ^f^	0.23 ^c^	37.95 ^b^	1.66 ^c^	0.01	0.08	0.04	19.71 ^f^	0.03	0.01 ^a^	0.30 ^b^	0.05	0.07	0.09 ^b^	0.01 ^a^	0.01 ^a^	0.01 ^a^	0.16 ^ab^
C	0.09 ^c^	0.05	2.71 ^c^	0.17 ^a^	20.68 ^a^	2.99 ^d^	0.03	0.02	0.00	17.19 ^e^	0.01	0.06 ^b^	0.24 ^a^	0.01	0.02	0.03 ^a^	0.02 ^a^	0.04 ^b^	0.02 ^a^	0.10 ^a^
D	0.02 ^a^	0.14	3.45 ^e^	0.16 ^a^	41.37 ^d^	1.52 ^b^	6.11	0.24	0.06	7.37 ^a^	1.56	0.23 ^c^	5.51 ^f^	0.01	0.00	0.24 ^c^	0.09 ^c^	0.06 ^c^	3.50 ^d^	0.48 ^c^
E	0.02 ^a^	0.05	2.48 ^b^	0.21 ^bc^	39.66 ^c^	1.19 ^a^	0.05	0.02	0.03	13.79 ^c^	0.42	0.19 ^c^	0.78 ^c^	0.03	0.11	0.01 ^a^	0.07 ^b^	0.01 ^a^	0.11 ^b^	0.32 ^bc^
F	0.07 ^c^	0.09	3.07 ^d^	0.16 ^a^	45.19 ^e^	3.77 ^e^	2.28	0.19	0.06	12.91 ^b^	1.07	0.46 ^d^	3.43 ^e^	0.06	0.00	0.08 ^b^	0.25 ^d^	0.01 ^a^	0.97 ^c^	0.85 ^d^
**Time After Opening (Months)**																				
0	0.11 ^c^	0.06	3.05 ^c^	0.21 ^b^	40.19 ^c^	2.45 ^a^	1.10	0.07	0.03	13.20 ^a^	0.38	0.10 ^a^	1.43 ^a^	0.04	0.04	0.09 ^a^	0.05 ^a^	0.03 ^a^	0.50 ^a^	0.32 ^ab^
3	0.06 ^b^	0.07	2.71 ^a^	0.15 ^a^	39.84 ^b^	2.70 ^b^	1.64	0.11	0.04	15.42 ^c^	0.58	0.16 ^b^	2.08 ^b^	0.05	0.04	0.14 ^c^	0.08 ^b^	0.03 ^a^	0.88 ^b^	0.41 ^b^
6	0.05 ^a^	0.06	2.78 ^b^	0.20 ^b^	39.28 ^a^	2.74 ^b^	1.78	0.12	0.05	15.32 ^b^	0.62	0.24 ^c^	2.09 ^b^	0.06	0.03	0.12 ^b^	0.09 ^c^	0.03 ^a^	0.97 ^c^	0.28 ^a^

Means with at least one of the same letters in the superscript (a, b, c, d, e, f) do not differ statistically at *p* ≤ 0.05 (for all columns separately); the main animal protein sources declared by the manufacturers were lamb (food A), poultry (food B), insects (food C), fish (foods D and F), and pork (food E).

**Table 5:** The average content of fatty acid groups and overall averages over time for all dog foods after opening the packages.

Dog Food	MUFA	PUFA	n-3	n-6	n-6/n-3	h/H	DFA	OFA	AI	TI
A	90.30 ^a^	27.78 ^a^	1.06 ^c^	17.38 ^d^	16.39 ^b^	5.55 ^f^	84.73 ^f^	13.85 ^a^	0.22 ^a^	3.75 ^b^
B	66.05 ^a^	30.49 ^a^	0.40 ^b^	19.89 ^e^	50.15 ^c^	2.26 ^c^	72.28 ^b^	26.23 ^e^	0.50 ^d^	2.29 ^a^
C	40.09 ^a^	26.44 ^a^	0.32 ^a^	17.25 ^d^	58.91 ^d^	0.78 ^a^	48.34 ^a^	50.39 ^f^	1.63 ^f^	2.28 ^a^
D	80.15 ^a^	26.19 ^a^	9.07 ^e^	7.77 ^a^	0.93 ^a^	2.91 ^d^	75.83 ^d^	18.93 ^c^	0.43 ^c^	29.3 ^f^
E	65.50 ^a^	23.28 ^a^	1.01 ^c^	13.93 ^c^	13.78 ^b^	2.10 ^b^	72.53 ^c^	25.85 ^d^	0.52 ^e^	4.47 ^c^
F	82.15 ^a^	28.45 ^a^	4.41 ^d^	13.35 ^b^	3.15 ^a^	3.62 ^e^	78.84 ^e^	17.28 ^b^	0.33 ^b^	14.19 ^d^
**Time After Opening (Months)**										
0	70.81 ^a^	23.58 ^a^	1.99 ^a^	13.41 ^a^	18.11 ^a^	2.27 ^a^	70.72 ^a^	26.90 ^c^	0.61 ^b^	7.25 ^a^
3	70.86 ^a^	28.79 ^a^	3.02 ^b^	15.73 ^c^	29.53 ^c^	3.21 ^c^	72.89 ^c^	24.52 ^a^	0.59 ^a^	10.27 ^b^
6	70.44 ^a^	28.95 ^a^	3.12 ^c^	15.65 ^b^	24.01 ^b^	3.13 ^b^	72.65 ^b^	24.84 ^b^	0.61 ^b^	10.55 ^c^

MUFA—monounsaturated fatty acids, PUFA—polyunsaturated fatty acids, h/H—hypocholesterolemic/hypercholesterolemic acids, DFA—hypocholesterolemic acids, OFA—hypercholesterolemic acids, AI—atherogenic index, TI—thrombogenic index; means with at least one of the same letters in the superscript (a, b, c, d, e, f) do not differ statistically at *p* ≤ 0.05 (for all columns separately); the main animal protein sources declared by the manufacturers were lamb (food A), poultry (food B), insects (food C), fish (foods D and F), and pork (food E).

**Table 6:** Pearson’s linear dependence coefficients.

Item	MUFA	PUFA	n-6	n-3	n-6/n-3	h/H	DFA	OFA	AI
PUFA	0.76 *p* < 0.000	1.00 *p* = ---	-	-	-	-	-	-	-
n-6	−0.17 *p* = 0.330	0.20 *p* = 0.240	1.00 *p* = ---	-	-	-	-	-	-
n-3	0.25 *p* = 0.146	0.03 *p* = 0.870	−0.81 *p* < 0.000	1.00 *p* = ---	-	-	-	-	-
n-6/n-3	−0.44 *p* = 0.007	0.10 *p* = 0.559	0.74 *p* < 0.000	−0.63 *p* < 0.000	1.00 *p* = ---	-	-	-	-
h/H	0.49 *p* = 0.002	0.13 *p* = 0.443	0.04 *p* = 0.832	0.19 *p* = 0.281	−0.49 *p* = 0.002	1.00 *p* = ---	-	-	-
DEFA	0.55 *p* = 0.001	0.09 *p* = 0.622	-0.16 *p* = 0.364	0.32 *p* = 0.059	−0.69 *p* < 0.000	0.84 *p* < 0.000	1.00 *p* = ---	-	-
OFA	−0.55 *p* < 0.000	−0.08 *p* = 0.627	0.26 *p* = 0.134	−0.43 *p* = 0.009	0.75 *p* < 0.000	−0.82 *p* < 0.000	−0.99 *p* < 0.000	1.00 *p* = ---	-
AI	−0.53 *p* = 0.001	−0.07 *p* = 0.683	0.19 *p* = 0.277	−0.32 *p* = 0.055	0.70 *p* < 0.000	−0.74 *p* < 0.000	−0.98 *p* < 0.000	0.98 *p* < 0.000	1.00 *p* = ---
TI	0.23 *p* = 0.171	0.02 *p* = 0.901	−0.82 *p* < 0.000	1.00 *p* < 0.000	−0.62 *p* < 0.000	0.16 *p* = 0.366	0.29 *p* = 0.081	−0.41 *p* = 0.014	−0.30 *p* = 0.073

MUFA—monounsaturated fatty acids, PUFA—polyunsaturated fatty acids, h/H—hypocholesterolemic/hypercholesterolemic acids, DFA—hypocholesterolemic acids, OFA—hypercholesterolemic acids, AI—atherogenic index, TI—thrombogenic index.

**Table 7:** Regression equations and coefficients of determination of observed and statistically documented dependencies of the analyzed parameters.

Correlation	Regression Equations	r^2^
MUFA vs. PUFA	PUFA = 9.427 + 0.250·MUFA	0.59
MUFA vs. n-6/n-3	n-6/n-3 = 49.272 − 0.359·MUFA	0.19
MUFA vs. h/H	h/H = 0.938 + 0.027·MUFA	0.24
MUFA vs. DFA	DFA = 56.456 + 0.221·MUFA	0.30
MUFA vs. OFA	OFA = 42.024 − 0.235·MUFA	0.31
MUFA vs. AI	AI = 1.231 − 0.009·MUFA	0.28
n-6 vs. n-3	n-3 = 12.207 − 0.636·n-6	0.66
n-6 vs. n-6/n-3	n-6/n-3 = −39.460 + 4.243·n-6	0.55
n-6 vs. TI	TI = 38.925 − 1.981·n-6	0.67
n-3 vs. n-6/n-3	n-6/n-3 = 36.236 − 4.555·n-3	0.39
n-3 vs. OFA	OFA = 29.790 − 1.612·n-3	0.18
n-3 vs. TI	TI = 1.001 + 3.078·n-3	1.00
n-6/n-3 vs. h/H	h/H = 3.666 − 0.033·n-6/n-3	0.24
n-6/n-3 vs. DFA	DFA = 80.281 − 0.343·n-6/n-3	0.48
n-6/n-3 vs. OFA	OFA = 16.184 + 0.387·n-6/n-3	0.56
n-6/n-3 vs. AI	AI = 0.265 + 0.014·n-6/n-3	0.49
n-6/n-3 vs. TI	TI = 15.611 − 0.262·n-6/n-3	0.38
h/H vs. DFA	DFA = 54.412 + 6.161·h/H	0.71
h/H vs. OFA	OFA = 43.495 − 6.301·h/H	0.68
h/H vs. AI	AI = 1.247 − 0.224·h/H	0.55
DFA vs. OFA	OFA = 10.44 − 1.041·DFA	0.98
DFA vs. AI	AI = 3.544 − 0.041·DFA	0.97
OFA vs. AI	AI = −0.378 + 0.039·OFA	0.96
OFA vs. TI	TI = 17.783 − 0.332·OFA	0.17

MUFA—monounsaturated fatty acids, PUFA—polyunsaturated fatty acids, h/H—hypocholesterolemic/hypercholesterolemic acids, DFA—hypocholesterolemic acids, OFA—hypercholesterolemic acids, AI—atherogenic index, TI—thrombogenic index.

**Table 8:** The average content of polyphenols and antioxidant properties over time and averages of all foods after opening the packages.

Dog Food	Polyphenols (mg CAE/100 g DM)	Polyphenols (mg GAE/100 g DM)	DPPH (µM Trolox/1 g DM)	FRAP (µM Trolox/1 g DM)	ABTS (µM Trolox/1 g DM)	RSA ABTS (%)
A	276.10 ^b^	137.20 ^b^	6.25 ^b^	29.76 ^d^	15.77 ^bc^	53.74 ^e^
B	196.30 ^a^	97.60 ^a^	4.57 ^a^	23.99 ^a^	10.31 ^a^	46.65 ^a^
C	359.90 ^d^	178.90 ^d^	7.67 ^cd^	32.78 ^e^	15.21 ^b^	51.83 ^b^
D	350.00 ^d^	17400 ^d^	6.70 ^bc^	26.19 ^b^	17.38 ^d^	53.72 ^d^
E	293.40 ^c^	145.80 ^c^	7.94 ^d^	27.93 ^c^	17.00 ^cd^	53.53 ^c^
F	1049.60 ^e^	521.70 ^e^	1202 ^e^	38.30 ^f^	22.58 ^e^	60.15 ^f^
**Time After Opening (Months)**						
0	382.30 ^b^	190.00 ^b^	9.73 ^c^	47.40 ^c^	16.29 ^b^	45.57 ^a^
3	361.00 ^a^	179.40 ^a^	5.78 ^a^	18.48 ^a^	14.09 ^a^	53.51 ^b^
6	519.40 ^c^	258.20 ^c^	7.07 ^b^	23.59 ^b^	18.74 ^c^	60.73 ^c^

Means with at least one of the same letters in the superscript (a, b, c, d, e, f) do not differ statistically at *p* ≤ 0.05 (for all columns separately); the main animal protein sources declared by the manufacturers were lamb (food A), poultry (food B), insects (food C), fish (foods D and F), and pork (food E).

## Data Availability

The original contributions presented in this study are included in the article/Supplementary Material. Further inquiries can be directed to the corresponding authors.

## Supplementary Materials

The following supporting information can be downloaded at: https://www.mdpi.com/article/10.3390/molecules30173524/s1, Table S1: Main components of analyzed dog foods.

## Abbreviations

The following abbreviations are used in this manuscript:

ABTS	2,2′-azinobis-(3-ethylbenzothiazoline-6-sulfonic acid)
AFR	adverse food reaction
AI	atherogenic index
ANOVA	two-way analysis of variance
BF3	boron trifluoride
BHA	butylated hydroxyanisole
BHT	butylated hydroxytoluene
CA	crude ash
CF	crude fiber
CGA	chlorogenic acid equivalents
CP	crude protein
DE	digestible energy
DFA	hypocholesterolemic acids
DPPH	2,2-diphenyl-1-picrylhydrazyl
ED	energy digestibility
EE	ether extract
FA	fatty acids
FRAP	ferric reducing antioxidant power
GAE	gallic acid equivalents
GC-FID	flame ionization detector
GE	gross energy
h/H	hypocholesterolemic/hypercholesterolemic acids
HSD	honestly significant difference
ME	metabolizable energy
MUFA	monounsaturated fatty acids
NFE	nitrogen-free extracts
OFA	hypercholesterolemic acids
ORAC	oxygen radical absorbance capacity
OTC	over-the-counter
PCA	principal component analysis
PUFA	polyunsaturated fatty acids
ROS	reactive oxygen species
RSA	radical scavenging activity
SFA	saturated fatty acids
TI	thrombogenic index
TPTZ	2,4,6-tris(2-pyridyl)-s-triazine
UFA	unsaturated fatty acids
