# Supplementary material for: Changes in the Fatty Acid Composition and Antioxidant Properties in Mono-Protein Commercial Dry Dog Foods During Storage
Source: Molecules. 2025 Aug 28;30(17):3524. doi: 10.3390/molecules30173524 (PMC12430719; doi:10.3390/molecules30173524)
Supplement: Supplementary file 1 [file molecules-30-03524-s001.zip › molecules-3814718-supplementary.pdf]

Table S1. Main components of analyzed dog foods

Dog food	Origin of the ingredient		Potential sources of		
	**animal**	**vegetable**	**phenolic compounds**	**animal fat**	**vegetable fat**
A	lamb	sweet potatoes, potatoes, peas	dried chicory root, blueberry, raspberries	NP	canola oil
B	poultry	pea starch, potato flakes, lentils	rosemary extract	poultry fat	sunflower oil
C	insects	oat, potatoes, corn, peas	seaweed	insect oil	NP
D	fish	peas, lentils, chickpeas	fresh cranberries, fresh blueberries, chicory root, turmeric, milk thistle, burdock root, lavender flower, marshmallow root, rose hips	pollack oil	cold-pressed sunflower oil
E	pork	peas, lentils, chickpeas	sea algae, fresh whole cranberries, fresh whole blueberries, chicory root, turmeric, milk thistle, burdock root, lavender flower, marshmallow root, rosehips	pork fat	NP
F	fish	sweet potatoes, lentils, tapioca, chickpeas	dried cranberry, chamomile powder, burdock root powder, anise and fenugreek, peppermint, calendula, grape seed extract, green tea extract, dried dandelion, dried blueberries, ginseng, thyme, marjoram, oregano, parsley, sage	NP	olive oil

NP – not provided by the manufacturer.
